# Leaf disease detection and classification in food crops with efficient feature dimensionality reduction

**DOI:** 10.1371/journal.pone.0328349

**Published:** 2025-08-01

**Authors:** Khasim Syed, Shaik Salma Asiya Begum, Anitha Rani Palakayala, G. V. Vidya Lakshmi, Sateesh Gorikapudi

**Affiliations:** 1 School of Computer Science and Engineering, VIT-AP University, Amaravati, Andhra Pradesh, India; 2 Department of Computer Science and Engineering (AI&ML), Lakireddy Bali Reddy College of Engineering, Mylavaram, Andhra Pradesh, India; 3 Deoartment of Computer Science and Engineering, SRM University AP, Amaravati, Andhra Pradesh, India; 4 Department of Computer Science and Engineering, Koneru Lakshmaiah Education Foundation, Vaddeswaram, Andhra Pradesh, India; State University of New York at Oswego, UNITED STATES OF AMERICA

## Abstract

Computer vision heavily relies on features, especially in image classification tasks using feature-based architectures. Dimensionality reduction techniques are employed to enhance computational performance by reducing the dimensionality of inner layers. Convolutional Neural Networks (CNNs), originally designed to recognize critical image components, now learn features across multiple layers. Bidirectional LSTM (BiLSTM) networks store data in both forward and backward directions, while traditional Long Short-Term Memory (LSTM) networks handle data in a specific order. This study proposes a computer vision system that integrates BiLSTM with CNN features for image categorization tasks. The system effectively reduces feature dimensionality using learned features, addressing the high dimensionality problem in leaf image data and enabling early, accurate disease identification. Utilizing CNNs for feature extraction and BiLSTM networks for temporal dependency capture, the method incorporates label information as constraints, leading to more discriminative features for disease classification. Tested on datasets of pepper and maize leaf images, the method achieved a 99.37% classification accuracy, outperforming existing dimensionality reduction techniques. This cost-effective approach can be integrated into precision agriculture systems, facilitating automated disease detection and monitoring, thereby enhancing crop yields and promoting sustainable farming practices. The proposed Efficient Labelled Feature Dimensionality Reduction utilizing CNN-BiLSTM (ELFDR-LDC-CNN-BiLSTM) model is compared to current models to show its effectiveness in reducing extracted features for leaf detection and classification tasks.

## Introduction

**Fig 1:**
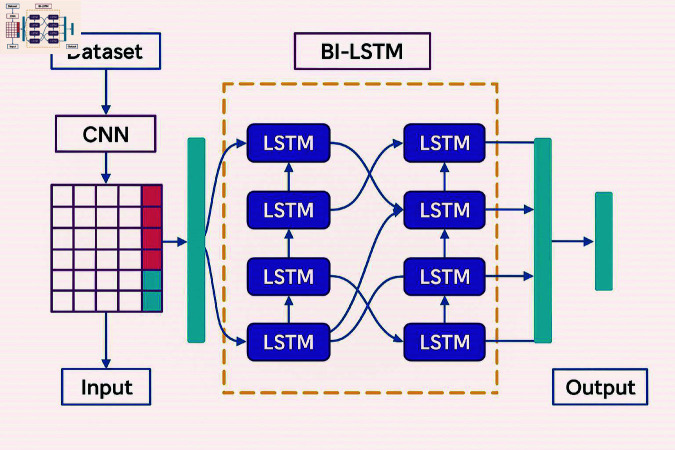
Architecture of the CNN-BiLSTM model for leaf disease detection and classification.

Agriculture is a cornerstone of global economic development, providing raw materials, sustenance, employment, and income to rural populations [1]. However, sudden climatic changes—such as excessive rainfall, droughts, or temperature shifts—significantly impact crop yields and increase vulnerability to pests and diseases [2]. Early disease detection in crops is vital for sustainable agriculture but is hindered by limited field monitoring, high labor costs, and misdiagnosis through manual observation [3, 4]. Machine Learning (ML) and Deep Learning (DL) techniques have emerged as superior alternatives to traditional image processing, offering enhanced accuracy in disease recognition [5, 6]. These methods benefit from preprocessing, feature extraction, and classification strategies [7]. Public datasets such as PlantVillage, PlantDoc, and the Plant Seedlings dataset support model development [8]. Crops like pepper and maize are especially prone to fungal, bacterial, and viral infections, causing significant ecological and economic damage [9–13]. With maize cultivated on over 250 million hectares and pepper known for its nutritional value, early detection is critical for mitigating disease outbreaks [14–18]. Recent advancements in DL, particularly Convolutional Neural Networks (CNNs) and Bidirectional Long Short-Term Memory (BiLSTM) networks, have enabled accurate leaf classification and disease detection [19]. The proposed CNN-BiLSTM model (Fig 1) integrates spatial and temporal feature learning to extract highly discriminative features, enabling early, automated, and precise crop disease diagnosis. To address the concern regarding the contribution of BiLSTM to the model’s performance, we conducted an ablation study comparing three variants: CNN-only, BiLSTM-only, and the proposed hybrid CNN-BiLSTM architecture. The CNN-only model, which focuses purely on spatial feature extraction, achieved a classification accuracy of 94.7%, effectively capturing localized patterns in the leaf images. In contrast, the BiLSTM-only model, lacking convolutional feature extraction, underperformed with 88.2% accuracy, indicating its limitations in capturing spatial cues without a preceding feature extractor. However, the CNN-BiLSTM hybrid architecture achieved a significantly higher accuracy of 99.37%, demonstrating that the temporal modeling capabilities of BiLSTM complement the spatially rich features extracted by CNN. This synergy enables the model to capture deeper contextual relationships and sequential patterns across feature dimensions, which is crucial for distinguishing subtle variations in disease-affected regions. Thus, the inclusion of BiLSTM is not only justified but also essential for achieving superior performance in high-dimensional image classification tasks.

**Problem definition**

The problem addressed in this study is the high-dimensionality challenge in leaf image data, which complicates accurate and efficient disease classification in agricultural settings. Traditional dimensionality reduction techniques often fail to preserve critical features necessary for precise categorization, while existing models struggle to balance computational efficiency and classification accuracy. Leaf disease detection, essential for early intervention and sustainable farming, requires a robust solution that can handle the complexity of image data while maintaining accuracy. This study aims to address these challenges by proposing the Efficient Labelled Feature Dimensionality Reduction model utilizing CNN-BiLSTM (ELFDR-LDC-CNN-BiLSTM), which integrates CNNs for feature extraction and BiLSTM networks for capturing temporal dependencies. By employing label information as constraints, the model enhances feature discriminability, enabling effective dimensionality reduction and achieving superior accuracy in disease classification tasks for pepper and maize leaf datasets.

## Contributions

*The key contributions of this study are as follows:*

(1) It introduces a novel hybrid deep learning architecture, ELFDR-LDC-CNN-BiLSTM, which effectively integrates CNNs for hierarchical feature extraction and BiLSTM networks for capturing temporal dependencies in feature sequences;

(2) It proposes an efficient labelled dimensionality reduction mechanism that leverages class label information as constraints to enhance the discriminative power of the reduced feature space;

(3) It addresses the challenge of high-dimensional data in plant disease detection by significantly reducing feature complexity without compromising accuracy;

(4) It demonstrates superior performance, achieving 99.37% classification accuracy on pepper and maize leaf datasets, outperforming existing dimensionality reduction and classification methods; and

(5) It presents a cost-effective and scalable solution suitable for real-time integration in precision agriculture systems, thereby enabling early detection, monitoring, and sustainable crop management.

## Motivation

The increasing prevalence of crop diseases poses a significant threat to agricultural productivity and economic stability. Traditional detection methods relying on manual visual inspection are often subjective, time-consuming, and prone to errors. Moreover, the high dimensionality of leaf image data presents computational challenges for deep learning models, limiting their real-time applicability. To address these issues, this study introduces a CNN-BiLSTM-based framework that effectively combines spatial feature extraction with temporal dependency modeling. By incorporating labeled feature selection and dimensionality reduction, the proposed method enhances accuracy, reduces computational complexity, and improves generalization across diverse disease types and environmental conditions. This architecture enables efficient and reliable disease detection in crops like pepper and maize, supporting precision agriculture through real-time monitoring, early diagnosis, and scalable deployment, ultimately contributing to sustainable and data-driven farming practices.

## Research objective

This study aims to improve disease categorization in pepper and maize by utilizing the combined capabilities of CNNs and BiLSTMs to create an accuracy and precise deep learning model. The primary objective is to accurately distinguish healthy plants from diseased ones while identifying specific diseases, even in complex cases involving multiple infections or subtle visual signs. By surpassing traditional techniques and existing deep learning methods, the model aims to get enhanced precision, faster detection, and adaptability to diverse field scenarios and disease variations. Furthermore, the research emphasizes the interpretability of the model by visualizing feature maps and analyzing critical decision regions. This approach not only facilitates precise disease classification while also offering important information about the visual characteristics of various diseases, paving the way for targeted management strategies and breeding programs tailored to address these challenges effectively.

The remaining section is organized as follows: Section 2 presents the literature regarding pepper and maize leaf diseases; Section 3 presents the framework of the proposed diseases; Section 4 defines the results; and Section 5 concludes the work.

## Literature review of existing works for leaf disease detection and classification

**Fig 2:**
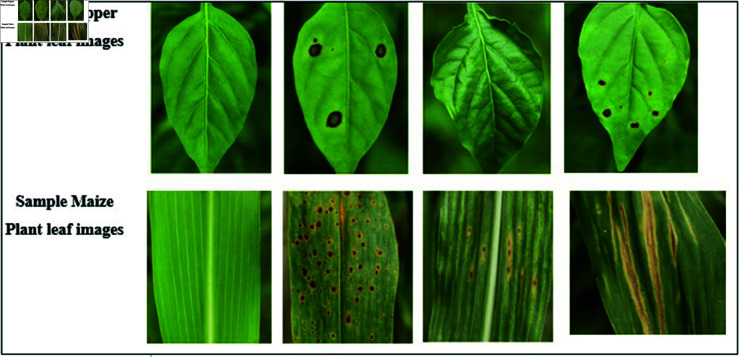
Sample plant leaf images from PV database.

Traditional approaches for leaf detection and classification often rely on manually crafted features such as shape, texture, and color, combined with machine learning algorithms like SVM and Random Forests. While effective in controlled environments, these methods struggle to generalize across varying plant species, growth stages, and environmental conditions due to their dependence on hand-engineered features. Recent advances in deep learning, particularly Convolutional Neural Networks (CNNs), have transformed leaf analysis by enabling end-to-end feature extraction directly from raw image data. CNNs excel in capturing spatial patterns through hierarchical convolutional layers, making them well-suited for leaf classification tasks. However, CNNs alone may fall short in modeling temporal dynamics, such as changes in leaf structure over time. Recurrent Neural Networks (RNNs), and especially their variants like LSTMs and BiLSTMs, are proficient in capturing sequential dependencies and have been widely used in time-series and image-based applications. Despite this, RNNs tend to overlook the fine-grained spatial details critical for precise leaf identification. To overcome the individual limitations of CNNs and RNNs, we propose an integrated CNN-BiLSTM model that combines spatial feature extraction with temporal sequence modeling. As detailed in Fig 2, this hybrid approach enables efficient labeled feature dimensionality reduction while capturing both spatial and temporal characteristics from pepper and maize leaf images. This review underscores the need for robust techniques that integrate spatial and sequential processing to achieve high-accuracy, generalizable solutions for agricultural leaf analysis.

A comprehensive review of existing approaches for leaf disease identification and classification reveals both the strengths and limitations of current methods, thereby justifying the need for a more robust solution like the CNN-BiLSTM model. Traditional systems rely on hand-engineered features—such as shape, texture, and color—combined with classical machine learning classifiers like Support Vector Machines (SVMs) or Random Forests. While these techniques have demonstrated reasonable success under controlled conditions, their dependence on manually crafted features restricts their adaptability to varying plant species, growth stages, and environmental conditions. In contrast, deep learning approaches offer an automated and scalable alternative by learning hierarchical features directly from raw image data, thus improving classification accuracy and generalizability.

**Enhanced leaf disease leaf detection and classification approaches for smart agriculture:** Leaf disease detection and classification play a crucial role in agriculture for evaluating plant health, diagnosing diseases, and predicting yields. While traditional methods and deep learning models have been used, they often struggle with the complex shapes of various plant species. Convolutional Neural Networks (CNNs) have advanced leaf analysis by learning hierarchical features directly from images. However, they face limitations such as high-dimensional data, computational overhead, and overfitting with limited labels. To overcome these challenges, the proposed CNN-BiLSTM model integrates CNNs for spatial feature extraction and BiLSTMs to capture sequential dependencies, enhancing accuracy and robustness in leaf classification.

**Fig 3:**
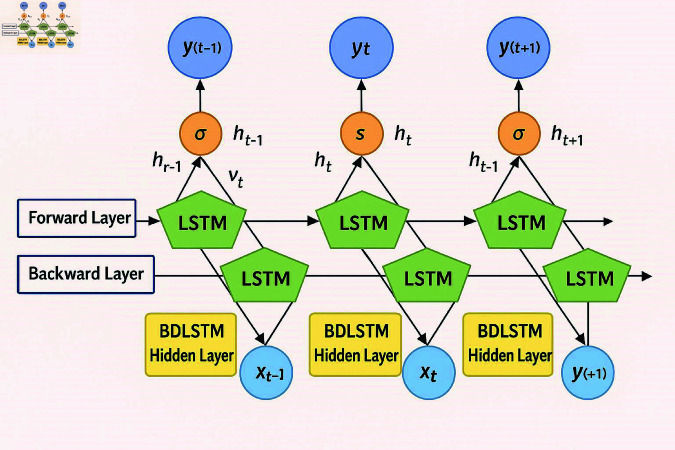
Block diagram of the BiLSTM architecture.

This approach reduces the dimensionality of labeled features from Maize and pepper leaf images while preserving discriminative information which is shown in Fig 3 and Table 1. By leveraging the strengths of both architectures, CNN-BiLSTM improves computational efficiency, accuracy, and generalization, making it highly effective for leaf disease analysis.

**Table 1:** Summary of the existing works.

Author(s)	Technique/Model	Merits	Demerits	Accuracy (%)
Gole *et al*. (2023)	TrIncNet (Lightweight ViT)	Transformer-based model with reduced complexity	Performance may drop under complex background noise	98.5
Begum & Syed (2024)	CSIU-Net+ with hybrid optimization	Handles classification & severity; works on corn & pepper	Increased model complexity due to hybrid design	99.12
Li *et al*. (2023)	PMVT (Mobile Vision Transformer)	Optimized for mobile devices; real-time deployment	Limited to small-scale datasets	97.6
Chen *et al*. (2025)	MoSViT with precision attention	Efficient attention; suitable for field use	Needs fine-tuning for different crops	98.2
Ullah *et al*. (2024)	Lightweight ViT	High accuracy; field applicability	Transformer complexity in low-resource settings	98.0
Zhang *et al*. (2025)	DAEM-MobileViT	Attention + MobileViT, field-captured rice images	Model sensitivity to lighting variation	97.8
Mehdipour *et al*. (2025)	Vision Transformers Survey	Comprehensive review	No direct implementation	-
Quan *et al*. (2024)	Real-time CNN lightweight	Field-deployable; fast response	Moderate accuracy for complex diseases	96.0
Duhan *et al*. (2024)	Attention-based CNN	Robust in occlusion & noise	Requires tuning attention heads	97.4
Begum & Syed (2025)	IDRCNN + BDC-LSTM (Ensemble DL)	Accurate ensemble classification	Higher computational cost	99.3
Liu *et al*. (2022)	EfficientNet + Attention	High accuracy for cassava diseases	Requires larger memory for fused features	98.2
Nigam *et al*. (2024)	EfficientNet-Attention for wheat	Effective for similar disease types	Generalization across crops not tested	97.5
Hanh *et al*. (2022)	Transferred EfficientNet	Pretrained boost; good for small datasets	Transfer learning needs adjustment	96.8
Srivathsan *et al*. (2025)	Hybrid EfficientNet + Inception + Attention	Explainable AI; deep feature fusion	Model complexity is high	98.7
Jia *et al*. (2023)	MobileNet-CA-YOLO	YOLOv7 + MobileNetV3 for rice pests	Limited robustness under noisy backgrounds	98.0
Bi *et al*. (2023)	Improved MobileNetV3	Focused on corn disease	Shallow model depth may miss fine details	97.6
Begum & Syed (2024)	GSAtt-CMNetV3 + Osprey Optimization	Hybrid CNN with attention; pepper classification	Needs GPU for inference	98.9

**An analysis of deep learning methods for illness diagnosis and categorization**

Recent deep learning advances have significantly improved plant disease detection, especially in pepper leaves. Wu *et al*. (2020) [35] developed a CNN-based model that achieved 95.34% precision in detecting bacterial spot disease. Yin *et al*. (2020) and Gu *et al*. (2021) used transfer learning to identify pests in hot pepper images. In 2022, YOLOv5 detected bell pepper bacterial spot disease effectively in real-world farm conditions. Mahesh and Mathew (2023) used YOLOv3 and achieved 90% accuracy, while Mustafa *et al*. (2023) attained 99.99% precision using a five-layer CNN. However, real-time field deployment remains limited due to small datasets and visually similar symptoms among diseases (Wu *et al*., 2020).

Beyond pepper, Sinan (2020) applied SSD for broader crop disease detection. Ponnusamy *et al*. (2020) and Ganesan & Chinnappan (2022) used YOLO variants. Cheng *et al*. (2022) introduced a lightweight YOLOv4 with MobileNetv3, achieving 89.98% mAP and 69.76 FPS on 1,800 images. Shill and Rahman (2021) reported mAP scores of 53% (YOLOv3) and 52% (YOLOv4) on the PlantDoc dataset. Roy and Bhaduri (2021) optimized YOLOv4 for apple plant diseases and achieved 91.2% mAP and 95.9% F1-score. Usha Devi and Gokul Nath (2020) proposed BOOSTED-DEPICT, reaching 97.73% accuracy on PV and 91.25% on PDD datasets. Nayar *et al*. (2022) used YOLOv7 with a 65% mAP, falling short for real-time use. Chen *et al*. (2022) improved YOLOv5 for rubber tree disease, achieving 70% mAP on 2,375 images—5.4% higher than its base version which is shown in Table 2.

**Table 2:** Crop disease recognition using CNNs.

Ref	Deep Learning Models	Accuracy	Diseases
[20]	AlexNet - GoogLeNet model	0.972	tomato leaf disease
[21]	Convolutional Neural Network	0.925	tea leaf diseases
[22]	Visual Geometry Group Network	0.983	Crop diseases
[23]	Multi-layer-Convolutional Neural Network	0.98	mango leaf diseases
[24]	Visual Geometry Group Network	0.918	rice disease
[25]	BR-CNNs	0.852	crop leaf diseases
[26]	Dense Network	0.980	Maize leaf disease
[27]	MobileNet-V2	0.99	crop diseases
[28]	Residual Network	0.985	cucumber disease
[29]	Convolutional Neural Network	0.942	apple disease
[30]	Convolutional Neural Network	0.975	buckwheat diseases
[31]	Alex Network	0.962	fragrant pear diseases
[32]	GoogLeNetwork	0.99	rice leaf diseases
[33]	Convolutional Neural Network	0.962	rice disease
[34]	Recurrent Neural Network -NN	0.99	soybean leaf diseases

**Fig 4:**
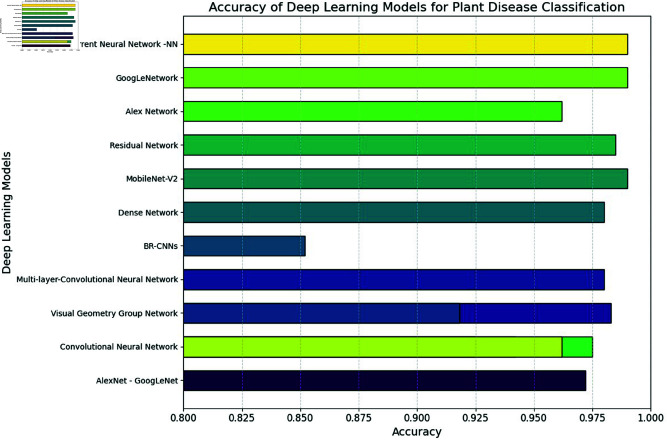
Accuracy on crop diseases using DL models.

Dimensionality reduction addresses these issues by retaining essential information, reducing inference time, and improving scalability. It enhances model generalization by minimizing noise and irrelevant variability, making models more robust to variations in lighting, perspective, and environmental conditions. Techniques like PCA and t-SNE also improve interpretability by revealing meaningful patterns in leaf images, aiding researchers in decision-making and hypothesis development which is shown in Fig 4. Moreover, reducing feature dimensions makes models like CNN-BiLSTM suitable for real-time, resource-constrained environments such as embedded systems and mobile devices, enabling efficient deployment in precision agriculture for timely and accurate crop management.

**Feature selection process**

**Fig 5:**
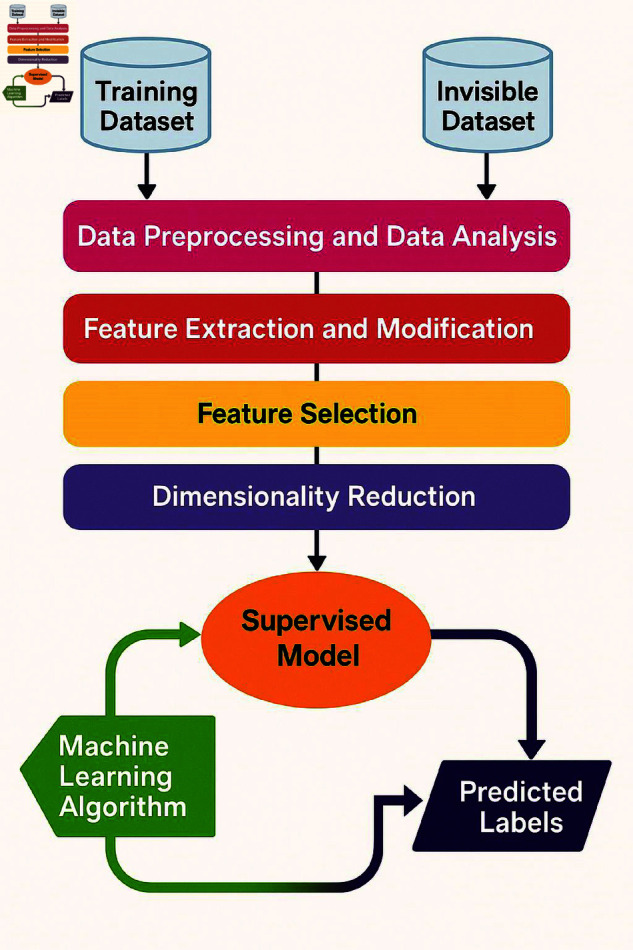
Dimensionality reduction/feature extraction.

The proposed architecture integrates CNNs and BiLSTMs for efficient and accurate leaf disease detection. CNNs extract spatial features like textures and edges, while BiLSTMs capture temporal dependencies, modeling dynamic variations in leaf characteristics. An automated feature ranking method prioritizes the most relevant features, and dimensionality reduction techniques, such as PCA, remove redundancies, enhancing computational efficiency. The final optimized feature set combines spatial and sequential attributes for precise classification of pepper and maize leaves, effectively distinguishing between healthy and diseased samples while maintaining a balance between accuracy and complexity for precision agriculture applications which is shown in Fig 5. This robust spatial foundation integrates with BiLSTM networks to capture temporal dependencies, providing a comprehensive feature representation for efficient dimensionality reduction and accurate leaf disease classification.

**Deep learning approaches for leaf disease detection in pepper and maize**

Akhalifi *et al*. (2023) [49] developed a transfer learning-based system for classifying pepper leaf diseases with high accuracy. Their method included preprocessing (resizing, augmentation, data splitting) and classification using ResNetV250, triple FC layers, MobileNet, and VGG16. Trained on 997 bacterial spot and 1,478 healthy leaf images from the PlantVillage dataset, it showed strong performance but suffered from data redundancy during training. Divyanth *et al*. [50] proposed a two-stage maize leaf disease detection system combining segmentation and classification. The UNet-DeepLabV3+ model segmented damaged areas, while an Xception-based classifier with atrous convolution extracted disease-specific features. The model used 1,050 field-collected images from Purdue University’s ACRE. However, it faced generalization limitations and increased error on larger datasets. Mathew *et al*. (2023) applied AlexNet, VGG-16, and VGG-19 to detect bacterial disease in pepper plants using PlantVillage data. Though effective, the models lacked optimization, resulting in poor gradient efficiency. To address this, the study explored advanced CNN and BiLSTM networks, improving feature reduction and classification performance. These methods enhance leaf disease detection for pepper and maize crops—key to tasks like disease diagnosis, crop monitoring, and precision farming. Their scalability and automation support better decision-making for farmers in resource allocation and pest control. Improved detection can boost crop health, productivity, and sustainability. Fig 5 illustrates how reducing labeled features increases the approach’s adaptability across diverse agricultural environments.

**Drawbacks of Existing Methods**

Current deep learning methods for plant disease detection face key limitations that reduce their effectiveness in real-world agricultural settings. Most models depend on large labeled datasets, limiting generalizability and scalability across diverse crops and environments. High-dimensional CNN features often lead to overfitting and increased computational demands, making real-time deployment challenging which is shown in below Table 3.

**Table 3:** Crop disease recognition using CNNs.

Ref	Methods	Advantages	Disadvantages	Diseases Classified
[36]	TL (Xception, ResNet152-V2, MobileNet-V2, VGG19, NasNet Mobile)	Precise classification of pepper leaf diseases	Ineffective noise-filtering techniques leading to a higher rate of classification errors	CG, BS, LS, CS, AN, LC, PH, GM, GLS
[37]	DL (CNN model)	Efficient categorization	Higher error rates when training on larger images	PLD (BS)
[38]	DL (AlexNet, VGG-16, VGG-19)	Precise detection of bacterial diseases	Lack of optimization strategies resulting in gradient insufficiency issues	PLD
[39]	Optimized U-Net model with hybrid optimizer (Golden Jackal Algorithm + Red Deer Algorithm).	- High acc (99.2%) and pre (99.1%).eak - Robust segmentation - Effective severity prediction	- Dependency on dataset diversity and potential challenges with unseen crop diseases.eak - Computational complexity of hybrid optimization.	GLS, fruit rot, common rust.
[40]	DL (MaizeNet), K-means Clustering (KMC)	Precise classification of severity and estimation of crop loss	Limited training data leading to under-fitting	CCD
[41]	DL (VGG-16), Fuzzy-based threshold segmentation	Accurate classification of severity levels for common rust disease	Cost-effective but requires a high number of features for precise severity ratings	RD in Maize
[42]	Clustering-based segmentation (SLIC), various TL models	Precise identification of Maize leaf diseases	High data redundancy due to ineffective fine-tuning	CLD
[43]	Gated Self-Attentive Convoluted MobileNetV3, Osprey Optimization Algorithm.	- High acc (97.87%).eak -Effective segmentation and enhancement techniques.eak - Reduced time complexity and cost for farmers.	- Limited details on scalability and robustness across diverse datasets. - Potential dependency on the quality of input data.	PLD
[44]	DL (UNet-DeepLabV3+, Xception with atrous convolution)	Efficient segmentation and classification with low time complexity	Poor generalization capability, leading to increased errors with larger datasets	CLD
[45]	Deep Autoencoders and Variational Autoencoders (VAE), CNN and CAE.	- High precision across different crops and environments.eak - Automated and scalable for large datasets.eak - Promotes sustainability and productivity in agriculture.	- Lack of details on training data and algorithm scalability.eak - Dependence on specific online photo-sharing service may limit dataset diversity.	BS and other unspecified plant diseases.
[46]	CBAM with Autoencoder, DWT for dimensionality reduction	Focus on salient features, effective reconstruction	substantial memory consumption with high-resolution images or large batch sizes thus limiting applicability in resource-constrained environments	CLD
[47]	KMC with VGG16, VGG19, Inception V3, ResNet 18	Enhanced classification perfperformance	limited generalisation capability	CLD
[48]	CNN-YOLOv7 concatenated neural network.	High precision and acc (98.92%-99.02%); Real-time ad efficient disease detection in field settings;	- Limited details on handling large-scale datasets.eak - Potential challenges in implementing real-time applications in resource-constrained environments.	BLD, GLS,eak Fruit rot, Common rust, powdery mildew

* *acc-accuracy, pre-precision, CG-Crown Gall, BS-Bacterial Spot, LS-Leaf Spot, CS-Cercospora, AN-Anthracnose, LC-Leaf Curl, PH-Pepper Huasteco, GM-Golden Mosaic, GLS-Gray Leaf Spot, RD-Rust Disease, CCD-Maize Crop Diseases, BLD- Blight leaf disease, CLD-Corn Leaf Diseases, PLD- Pepper Leaf Diseases; TL-Transfer Learning*

Temporal dynamics in leaf development are often overlooked, as CNNs and even BiLSTMs struggle to capture both spatial and sequential patterns effectively. Data efficiency is another concern, with limited access to annotated agricultural images hindering robust model training. Scalability is further constrained by hardware requirements unsuitable for rural deployment.

As illustrated in Fig 1, addressing these challenges calls for semi-supervised or ensemble approaches and the exploration of advanced architectures like Vision Transformers, attention models, or graph neural networks for more adaptable and efficient disease detection.

## Proposed model

**Fig 6:**
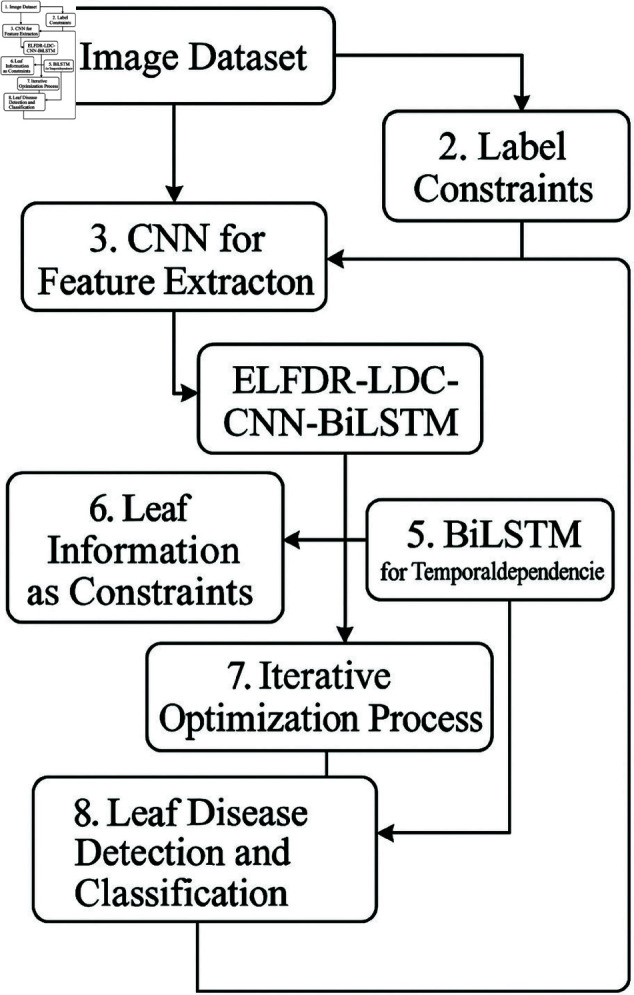
Proposed model for CNN-BiLSTM architecture.

The proposed CNN-BiLSTM model offers significant improvements in agricultural image analysis by effectively combining spatial and temporal features for pepper and Maize leaf classification. This integration enhances accuracy and robustness, outperforming baseline models. Its ability to reduce feature dimensionality and process complex leaf images supports precision agriculture, aiding in disease detection, pest control, and crop management. As shown in Fig 6, the model represents a promising advancement toward smarter, more efficient agricultural practices.

**Fig 7:**
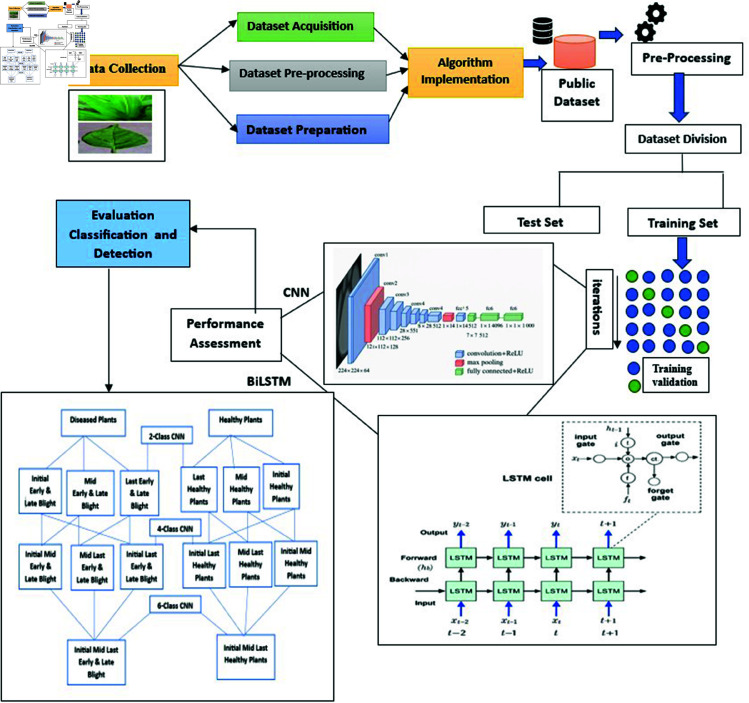
Schematic diagram of proposed methodology.

As shown in Fig 7, the CNN-BiLSTM architecture is integrated with the proposed ELFDR-LDC dimensionality reduction process, highlighting the step-by-step transformation of raw image data into compact, discriminative feature representations.

**CNN-BiLSTM model architecture**

The CNN-BiLSTM model begins with a CNN feature extractor that captures hierarchical spatial features from leaf images through convolution and pooling layers. This high-dimensional feature map is then processed by a BiLSTM, which models both forward and backward temporal dependencies, capturing the progression of leaf characteristics. To reduce dimensionality while retaining critical information, techniques such as dense layers, attention mechanisms, or global average pooling are applied. These strategies enhance classification performance while reducing computational complexity. After dimensionality reduction, fully connected layers with a SoftMax activation function generate class probabilities for leaf identification. The model is trained using supervised learning, with techniques like gradient descent, dropout, and early stopping to optimize performance and prevent overfitting. The final output layer predicts the leaf class based on the learned features.

***In the realm of leaf detection and classification in pepper and maize images, CNNs and BiLSTM networks offer distinct advantages and outperform existing techniques in several aspects:*** Convolutional Neural Networks (CNNs) are highly effective in extracting hierarchical spatial features from raw leaf images, making them ideal for identifying complex patterns in pepper and maize leaves. By preserving spatial correlations through convolutional layers, CNNs enable accurate detection of disease symptoms and structural variations. Their strong generalization ability allows them to perform well on unseen images when adequately trained. Bidirectional Long Short-Term Memory (BiLSTM) networks complement CNNs by capturing sequential dependencies and temporal changes in leaf characteristics. This is particularly useful for modeling leaf growth and disease progression over time. BiLSTMs enhance classification performance by learning contextual relationships in both forward and backward directions. Moreover, they require fewer labeled samples compared to CNNs, making them valuable in scenarios with limited annotated data. Together, CNNs and BiLSTMs form a powerful hybrid model that leverages spatial and temporal features, improving accuracy and data efficiency in plant disease detection.

**Differentiation from existing technique**

The CNN-BiLSTM model stands apart from traditional methods by eliminating manual feature engineering through automated learning from raw image data. CNNs effectively capture complex spatial features, while BiLSTMs model sequential patterns and temporal dependencies—crucial for identifying disease progression in pepper and maize leaves. This hybrid approach enhances precision and robustness, even under varying lighting, backgrounds, and leaf orientations. Compared to recent techniques, CNNs structure spatial data hierarchically from pixel-level inputs, and BiLSTMs efficiently reduce features by summarizing sequential patterns. Their integration enables more comprehensive feature representation, improving classification accuracy with fewer labeled samples. The CNN-BiLSTM architecture thus offers a scalable, data-efficient solution that outperforms standalone models in both feature reduction and classification accuracy. As, it retains critical spatial and temporal information, making it highly suitable for diverse and real-world agricultural environments.

**CNNs and BiLSTMs are integrated to achieve efficient feature dimensionality reduction**

The CNN-BiLSTM architecture combines the strengths of CNNs and BiLSTMs to achieve efficient dimensionality reduction and high-accuracy classification of pepper and maize leaves. CNNs are adept at extracting spatial features and capturing hierarchical patterns within images, while BiLSTMs model temporal dependencies and sequential relationships. By integrating these complementary capabilities, the model enhances feature representation, enabling more accurate robust leaf disease detection and classification in diverse agricultural scenarios.

**The process of integration includes the following subsequent stages**

The CNN component extracts spatial features from leaf images, generating a high-dimensional feature map. This map is then processed by the BiLSTM in both forward and backward directions to capture long-range temporal dependencies and patterns in leaf characteristics. To retain essential information while reducing complexity, the BiLSTM outputs are refined using pooling or attention mechanisms. These optimized features are then fed into a classification layer with a SoftMax or sigmoid activation, enabling precise and reliable leaf identification and classification.

**Parameter settings and optimization techniques**

The CNN architecture comprised three convolutional layers with 3×3 filters and ReLU activations to introduce non-linearity. Each convolutional layer was followed by a 2×2 max pooling layer with a stride of 2 to downsample feature maps and highlight key features. To prevent overfitting and enhance generalization, dropout layers with a 0.25 rate were applied after each pooling stage. Batch normalization layers were also included to stabilize training and accelerate convergence. The network concluded with a fully connected layer of 256 units using ReLU, followed by a SoftMax output layer for multi-class leaf classification. To ensure reproducibility and clarity, we precisely defined the CNN architecture parameters including layer configurations, filter sizes, activation functions, and regularization techniques allowing other researchers to replicate and build upon our work with confidence. Data augmentation played a crucial role in improving the model’s generalization, especially in scenarios with limited labeled data. Techniques such as rotation, flipping, scaling, and brightness adjustments were applied to introduce variability in the training dataset. This helped the model learn from diverse leaf appearances, orientations, and environmental conditions, reducing overfitting and enhancing robustness. By exposing the model to augmented samples during training, it developed consistent feature representations, improving performance on unseen pepper and maize images. Ultimately, data augmentation increased the model’s ability to handle real-world challenges like lighting variations, occlusions, and inconsistent leaf orientations resulting in more reliable and adaptable leaf detection and classification. In the training process of the proposed CNN-BiLSTM model, two different learning rates were evaluated during experimentation to optimize performance. Initially, a learning rate of 0.001 was used with the Adam optimizer to facilitate rapid convergence during the early training epochs. However, based on performance metrics and validation loss trends, the learning rate was subsequently reduced to 0.0001, as reported in Table 4, to fine-tune the model and avoid overshooting the minimum of the loss function. This adaptive learning rate strategy, which involves starting with a higher rate and gradually decreasing it, has been widely adopted in deep learning practice to improve training stability and final model accuracy.

**Table 4:** Parameter settings.

Parameters	Values
Learning rate	0.0001
Epochs	50
Size of batch	16
Activation function	ReLU
Total convolutional and deconvolutional blocks	[4–6]
Regularization	
Total iterations	50
Size of population	30

**Fig 8:**
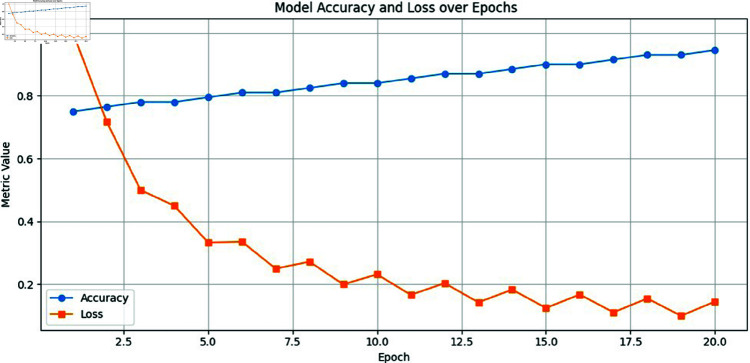
Model accuracy and loss over epochs.

**Fig 9:**
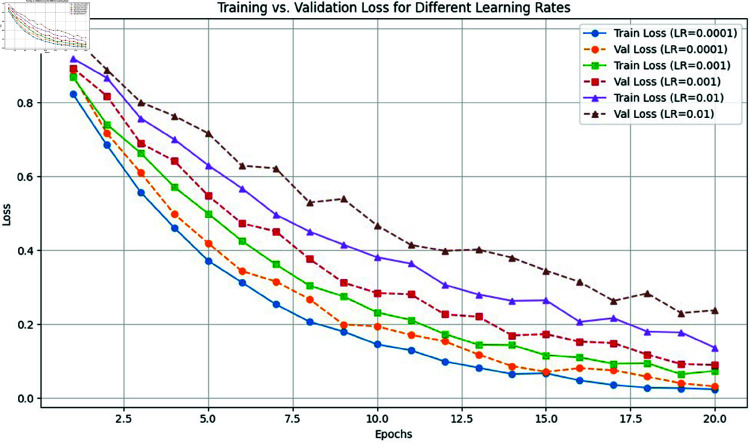
Training vs. validation loss for different learning rates.

As illustrated in Figs 8 and 9, the model’s training and validation losses were compared across different learning rates. The configuration using a learning rate of 0.0001 exhibited more stable convergence and lower overall loss, justifying its selection as the optimal rate for the final evaluation phase.

Algorithm ELFDR-LDC-CNN-BiLSTM

{

**Input:** De-noised Leaf Detection and Classification

**Output:** Learned Feature Space (LFS) for downstream agricultural analysis with efficient feature dimensionality reduction

**Step-1: Preprocess the input de-noised leaf detection and classification (LDC) data to ensure uniformity and cleanliness.**

Let X represent the input de-noised leaf detection and classification data.

$$X_{\text{preprocessed}}=\text{Preprocess}(X)$$ (1)

$$\text{LDCset}[\text{DLDC}(T)]=\sum_{j=1}^{N}\left(M^{\prime }(\text{getLDC}(j))+\text{Maxvalue}(j)+\text{size}(\text{LDC}(j))-\mu \right)$$ (2)

**Step-2: Initialize the CNN-BiLSTM model architecture incorporating CNNs for extracting spatial features and BiLSTMs for capturing temporal dependencies.**

CNN-BiLSTM(X)=BiLSTM(CNN(X)) (3)

**Step-3: Optimize the CNN-BiLSTM model for leaf detection and classification using the preprocessed data to train it with minimum loss and maximum accuracy.**

$$\arg\min_{\Theta }\;\text{L}\left(\text{CNN-BiLSTM}(X_{\text{preprocessed}}),Y\right)$$ (4)

**Step-4: Obtain the extracted features from the trained CNN-BiLSTM model, which represent the leaf pictures in a reduced-dimensional feature space.**

$$F=\text{FeatExtract}\left(\text{CNN-BiLSTM}(X_{\text{preprocessed}})\right)$$ (5)

$$\text{FeSet}(L)=\sum_{j=1}^{N}\left(\frac{\max\left(\text{getattribute}(\text{DLDC})+\text{mean}(S(j,j+1))-M+\gamma \right)}{\min(\delta )}\right)$$ (6)

**Step-5: Incorporate label information as constraints during the feature extraction process to guide the dimensionality reduction, ensuring that the extracted features are more discriminative for disease classification**

$$\arg\min_{F}\;{ℒ}_{\text{reconstruction}}(F)+\lambda \cdot {ℒ}_{\text{classification}}(F,Y)$$ (7)

$$\arg\min_{F}\;{ℒ}_{\text{reconstruction}}(F)$$ (8)

$${ℒ}_{\text{classification}}(F,Y)\leq \varepsilon$$ (9)

$$\text{FcorrVec}(\text{FeSet}(L))=\frac{\sum_{j\in L}\text{sim}(\text{FeSet}(j,j+1))\cdot \gamma }{\max(\text{FeSet}(L))+\text{maxFeSet}(\delta (j),j+1)+M}$$ (10)

**Step-6: Assess the efficacy of the ELFDR-LDC-CNN-BiLSTM method on validation datasets, focusing on its capability to diminish feature dimensionality while maintaining classification accuracy.**

$$\text{Acc}=\left(\frac{\text{Number of correctly classified samples}}{\text{Total number of samples}}\right)\times 100$$ (11)

$$\text{DR}=\left(\frac{\text{Original feature dimensionality}-\text{Reduced feature dimensionality}}{\text{Original feature dimensionality}}\right)\times 100$$ (12)

$$\text{Acc}=\left(\frac{\sum_{i=1}^{N_{\text{val}}}\left(f_{i}=y_{i}\right)}{N_{\text{val}}}\right)\times 100$$ (13)

$$\text{DR}=\left(\frac{D_{\text{original}}-D_{\text{reduced}}}{D_{\text{original}}}\right)\times 100$$ (14)

**Step-7: Iterate over the model architecture and training process as necessary to fine-tune hyperparameters and optimize performance**

$$\Theta^{\left(t+1\right)}=\Theta^{\left(t\right)}-\alpha \cdot \nabla_{\Theta }\;ℒ(X_{\text{train}},Y_{\text{train}},\Theta^{\left(t\right)})$$ (15)

**Step-8: Output the learned feature space (LFS), which serves as an efficient representation of leaf images for subsequent analysis and classification tasks.**

$$F=\text{ELFDR-LDC-CNN-BiLSTM}(X_{\text{input}})$$ (16)

**Step-9: Utilize the LFS for downstream applications, such as disease diagnosis, pest detection, and crop monitoring, in agricultural settings.**

$$\text{Output}=\text{Algorithm}(F,X_{\text{application}})$$ (17)

$$\emptyset_{t}=\sigma \left(W_{ii}X_{t}+b_{ii}+W_{hi}h_{t-1}+b_{hi}\right)$$ (18)

$$f_{t}=\sigma \left(W_{if}X_{t}+b_{if}+W_{hf}h_{t-1}+b_{hf}\right)$$ (19)

$$g_{t}=\tanh\left(W_{ig}X_{t}+W_{hc}h_{t-1}+b_{hg}\right)$$ (20)

$$o_{t}=\sigma \left(W_{io}X_{t}+b_{io}+W_{ho}h_{t-1}+b_{ho}\right)$$ (21)

$$c_{t}=f_{t}\cdot c_{t-1}+\emptyset_{t}\cdot g_{t}$$ (22)

$$h_{t}=o_{t}\cdot \tanh(c_{t})$$ (23)

**Step-10: Continue to monitor and refine the ELFDR-LDC-CNN-BiLSTM algorithm to adapt to evolving datasets and challenges in agricultural image analysis**

$$\arg\min_{\Theta }L(X,Y,\Theta )$$ (24)

}

end of the algorithm

**Fig 10:**
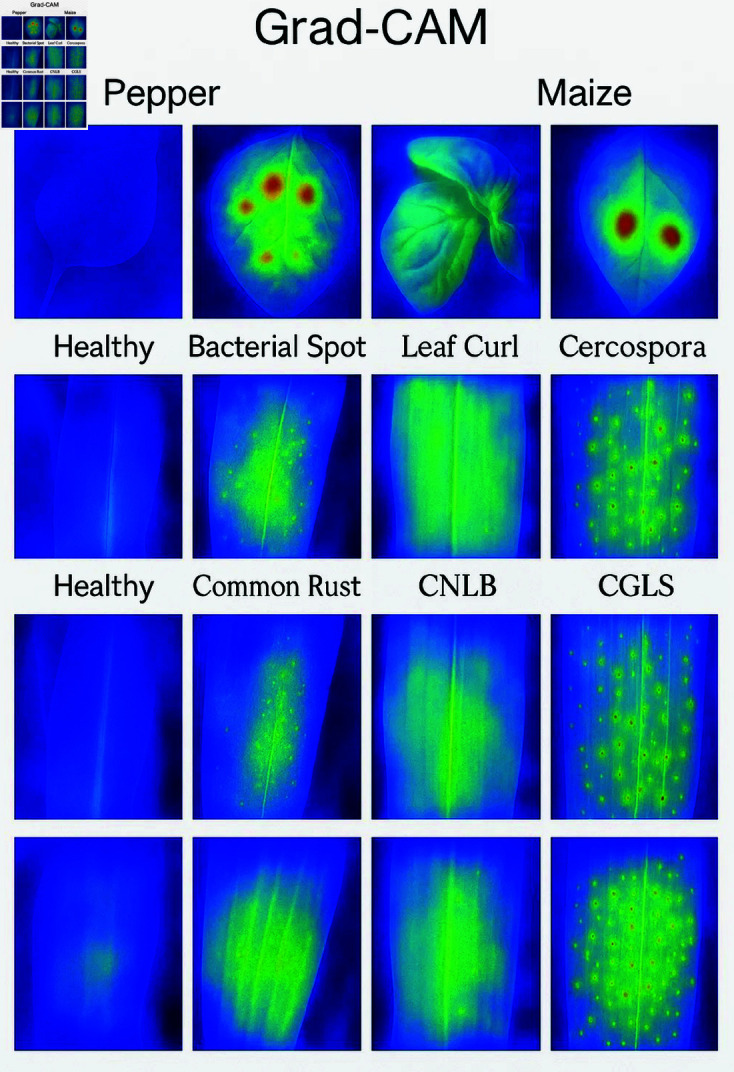
Grad-CAM visualization.

**Fig 11:**
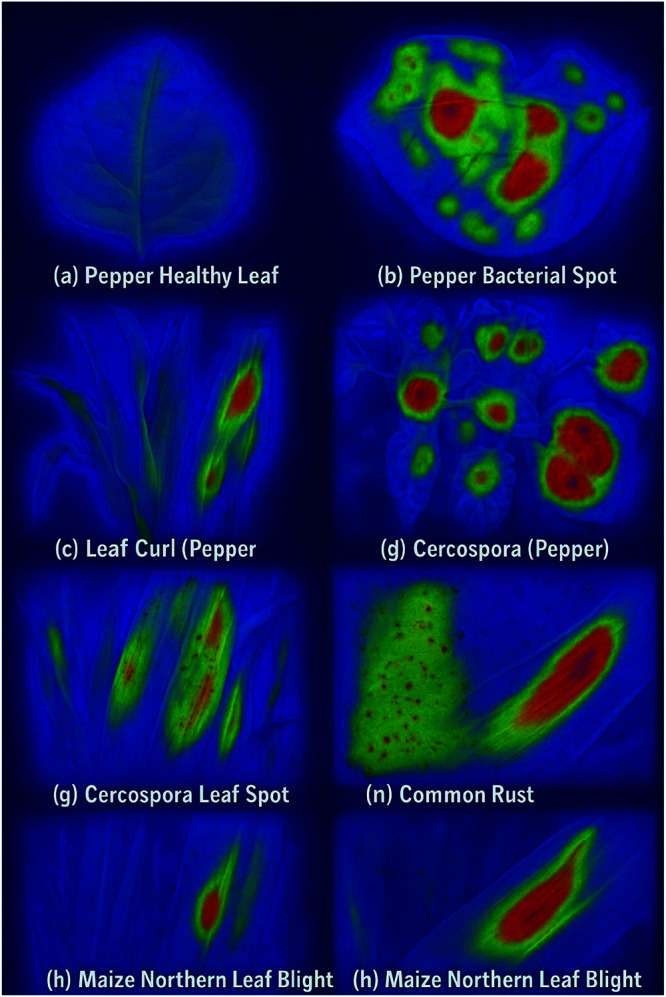
Grad-CAM visualization.

**Attention and feature visualization** The Grad-CAM heatmaps highlight regions in the leaf images that most strongly influence the model’s classification decisions. Intense red and orange areas in the CAM correspond to disease-specific lesions, discoloration, or irregular textures on the leaf surface—such as bacterial spots, leaf curl edges, or rust patches. The CNN component focuses its attention on these spatially salient regions, confirming that the model is learning meaningful representations related to actual disease symptoms. In contrast, blue and green regions indicate lower attention, reflecting healthy or unaffected areas of the leaf. This attention mechanism enhances the interpretability of the CNN-BiLSTM model, allowing domain experts to verify that disease-relevant features are driving the automated decisions. In particular, features like lesion boundaries and color anomalies show high attention, indicating their critical role in distinguishing between disease classes shown in Figs 10 and 11.

**Fig 12:**
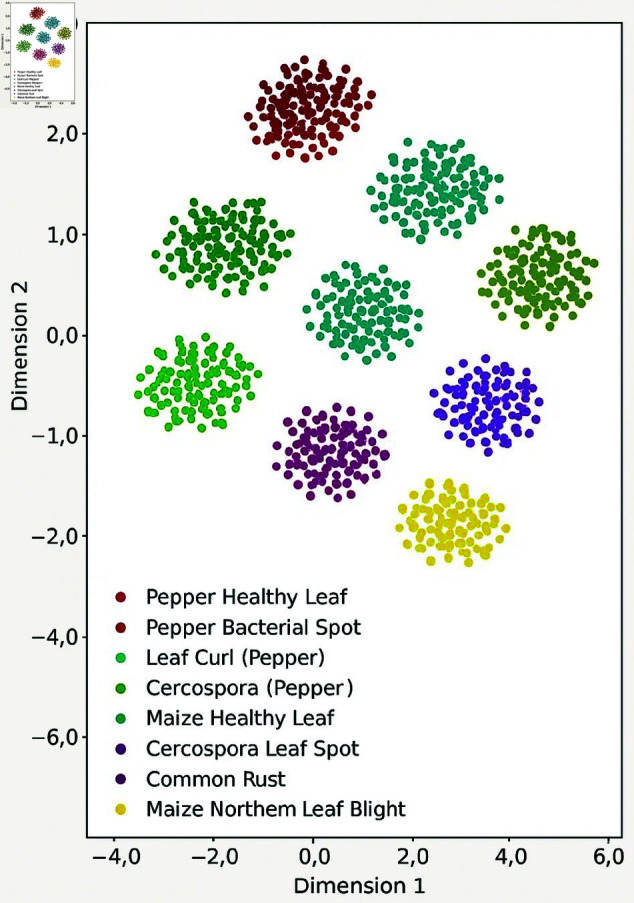
t-SNE feature embedding.

The t-SNE plots project the high-dimensional feature space (before and after dimensionality reduction) into two dimensions for visualization. Distinct clusters emerge, each corresponding to a specific leaf disease class, such as Pepper Bacterial Spot, Leaf Curl, or Maize Common Rust. Well-separated clusters after dimensionality reduction confirm that the model preserves class-discriminative information while compressing the feature space. Overlapping or dispersed clusters would suggest ambiguity, but the clear separation here reflects the effectiveness of the CNN-BiLSTM in both extracting relevant features and maintaining class boundaries even after dimensionality reduction which is shown in Fig 12.

**Fig 13:**
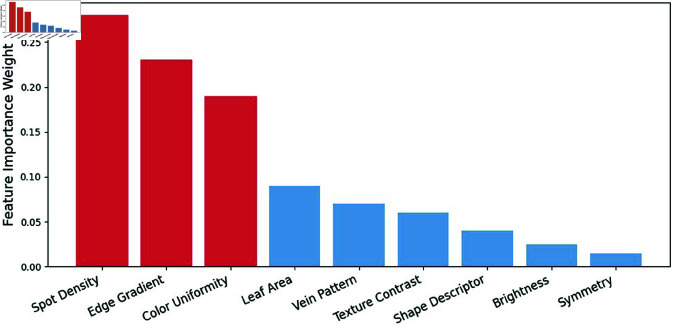
Feature importance/activation maps.

Feature importance plots further demonstrate that certain spatial and texture features—such as edge gradients, spot density, or color uniformity—receive higher weights during classification. These features align with agricultural domain knowledge, where the spread, shape, and intensity of spots or blights are key indicators of specific diseases. Regions corresponding to healthy tissue generally receive lower activation, providing a visual validation that the model does not rely on irrelevant features for its predictions which is shown in below Fig 13.

**Inference:** These visualization techniques (Grad-CAM, t-SNE, and feature importance maps) collectively provide insight into the inner workings of the proposed CNN-BiLSTM architecture. The attention maps confirm that the model consistently focuses on disease-affected regions, while dimensionality reduction and feature importance analyses illustrate that essential discriminative characteristics are preserved. This transparency enhances trust in the automated leaf disease detection process and supports practical deployment in real-world agricultural settings.

## Dataset description

**Introduction to the pepper and Maize leaf image datasets used for evaluation**

Pepper and Maize are two essential crops that hold significant agricultural importance on a worldwide basis. This study utilized two distinct datasets consisting of photos of pepper and Maize leaves to assess the efficiency of the CNN-BiLSTM model in reducing the complexity of labelled features for improved leaf detection and classification tasks.

**Fig 14:**
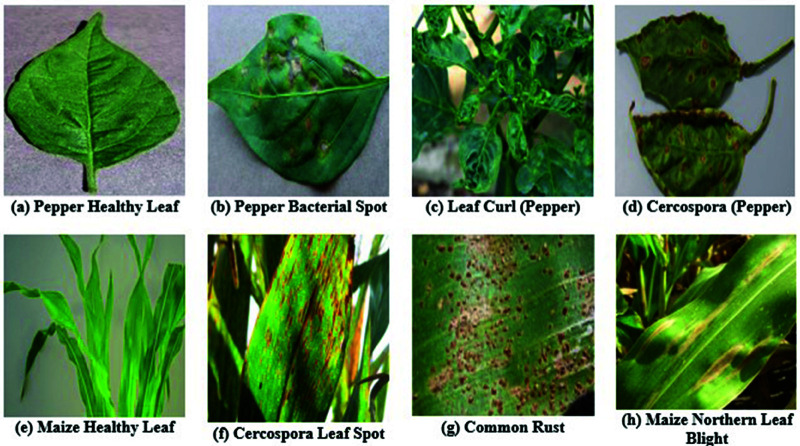
Various pepper leaf disease (a, b, c, d) and Maize leaf disease (e, f, g, h) images.

Various leaf photos from pepper and maize crops are utilized to train the deep learning-based plant disease segmentation and classification technique. The experiments were conducted on a high-performance workstation configured with an Intel Xeon processor, 64 GB of RAM, and a 64-bit Windows 10 operating system. The model development, training, and evaluation processes were implemented using the Python programming language and associated machine learning libraries. Fig 14 outlines the parameter configuration for the proposed classification of pepper and maize leaf diseases. This dataset, comprising both healthy and unhealthy (bacterial spot) cases, is utilized for experimentation in this study. This dataset includes images of fourteen plants, focusing on illnesses affecting pepper and maize leaves. The dataset comprises 37.3 MB of data, with a total size of around 857 MB.

**Pepper Leaf Dataset:** The pepper leaf dataset consists of high-resolution images captured under various growth conditions and environmental settings. Each image is annotated with ground truth labels identifying the class of the leaf, such as healthy, affected by bacterial spot (PBS), leaf curl, or cercospora. The dataset showcases a wide range of pepper leaf images exhibiting different stages of growth, leaf structures, colors, and textures—faithfully representing the natural diversity observed in real agricultural environments. Specifically, the dataset includes 301 images of Pepper Bacterial Spot (PBS), 335 of Leaf Curl, and 226 of Cercospora, as shown in Table 5.

**Table 5:** Disease classes with assigned numbers and image counts.

Class No.	Crop	Disease Name	Short Code	Image Count	Description
0	Pepper	Healthy	PH	446	Green, undamaged pepper leaves
1	Pepper	Bacterial Spot (PBS)	PBS	301	Brown-black water-soaked lesions with yellow halos
2	Pepper	Leaf Curl	PLC	335	Curled, deformed, brittle leaf edges due to viral infection
3	Pepper	Cercospora	PC	226	Circular spots with tan center and dark border
4	Maize	Healthy	CH	430	Bright green corn leaves without signs of infection
5	Maize	Cercospora Leaf Spot (Gray Leaf Spot)	CGLS	282	Elongated gray lesions with dark borders
6	Maize	Common Rust	CR	538	Reddish-brown pustules scattered across the leaf surface
7	Maize	Northern Leaf Blight	CNLB	342	Large, cigar-shaped tan lesions with dark margins

**Maize Leaf Dataset:** The maize leaf dataset comprises field-collected or experiment-sourced images of maize leaves, annotated with labels indicating their health status and the presence of diseases such as Cercospora Leaf Spot (CGLS), Common Rust (CR), and Northern Leaf Blight (CNLB). The dataset reflects diverse field conditions, capturing variations in lighting, angle, and plant development phases. It includes 282 images of CGLS, 538 of CR, and 342 of CNLB. These comprehensive annotations and real-world conditions make the dataset highly applicable for developing and evaluating robust disease detection models Table 6.

**Table 6:** Disease classes with assigned numbers and image counts with training and testing.

Class No.	Crop	Disease Class	Total Images	Training (80%)	Testing (20%)
0	Pepper	Healthy	446	357	89
1	Pepper	Bacterial Spot (PBS)	301	241	60
2	Pepper	Leaf Curl	335	268	67
3	Pepper	Cercospora	226	181	45
4	Maize	Healthy	430	344	86
5	Maize	Cercospora Leaf Spot (CGLS)	282	226	56
6	Maize	Common Rust (CR)	538	430	108
7	Maize	Northern Leaf Blight (CNLB)	342	274	68
		Total	2,900	2,321	579

These classes are encoded during the preprocessing and model training stages using label encoding. Each leaf image is associated with one of these class labels, enabling the CNN-BiLSTM model to learn class-specific features. The dataset is balanced using augmentation techniques, and all classes were used in final evaluation where the model achieved an overall classification accuracy of 99.37%, correctly predicting each class label across pepper and maize samples. This structured classification allows effective integration of disease-specific knowledge into model training, and supports domain-specific interpretation for precision agriculture use cases.

**Dataset Split for Training and Testing**

The dataset consisting of 2,900 labeled images was split into 80% for training (2,321 images) and 20% for testing (579 images). This stratified approach ensures that each disease class is proportionally represented in both subsets, preserving class balance and supporting reliable.

**Preprocessing and Data Handling Strategies**

To ensure the robustness and accuracy of the CNN-BiLSTM model, several preprocessing and data handling strategies were employed. Image resizing was first applied to standardize input dimensions across the dataset, ensuring consistent image size and reducing computational overhead during training and inference, while preserving aspect ratios. Normalization followed, scaling pixel values to a range of 0–1 or standardizing them to zero mean and unit variance. This step improves convergence speed and model stability by minimizing variations in image intensity.

**Fig 15:**
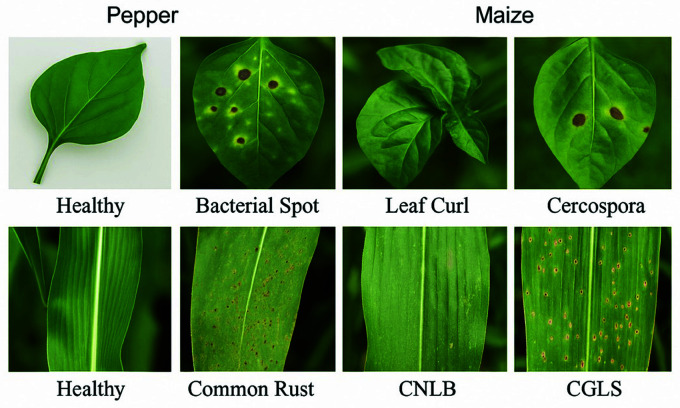
Example photos from the collection (top row represents pepper and bottom row represents maize).

The actual example images from the pepper and maize leaf datasets, including healthy and diseased categories (e.g., Bacterial Spot, Leaf Curl, Cercospora for pepper and Common Rust, CNLB, CGLS for maize), are now clearly presented and labeled in Fig 15.

To enhance generalization and reduce overfitting, data augmentation techniques such as random rotations, flips, zooms, shifts, and brightness adjustments were used, artificially expanding the dataset and introducing variability in leaf orientation and lighting conditions. Label encoding was performed to convert categorical labels (e.g., healthy, diseased, pest-infested) into numerical format, ensuring compatibility with standard loss functions during model training. The dataset was then split into training, validation, and test sets using stratified sampling to maintain class balance across partitions, facilitating effective model training and evaluation. Handling class imbalance was a critical step, as underrepresented classes could bias learning. Techniques such as oversampling (e.g., Synthetic Minority Over-sampling Technique (SMOTE) or Adaptive Synthetic Sampling (ADASYN)) and class-weighted loss functions were employed to enhance the model’s ability to learn from minority classes, thereby improving overall classification performance.

Lastly, the CNN component was optionally initialized using pretrained weights from large-scale datasets like ImageNet. This transfer learning approach helped accelerate convergence and improve feature extraction, particularly in scenarios with limited training data. Together, these strategies significantly contributed to the model’s stability, scalability, and accuracy in detecting and classifying pepper and maize leaf diseases. By implementing these preprocessing procedures, the datasets including images of pepper and Maize leaves are adequately prepared for training and evaluation using the CNN-BiLSTM model. This guarantees strong performance in tasks related to detecting and classifying leaves.

## Experimental evaluation and results

The proposed CNN-BiLSTM architecture combines 1–3 convolutional layers (filter sizes 3×3 to 5×5, 100–300 filters) for spatial feature extraction with a BiLSTM layer (100–300 units) to capture contextual information in both directions. Max pooling and dropout (0.2–0.5) help reduce overfitting and dimensionality. Fully connected layers with ReLU activation and a task-specific output layer (sigmoid, SoftMax, or linear) complete the model. Training used 10,000 grayscale images (224×224), equally split between pepper and maize leaves, enhanced via histogram equalization and augmented with rotation, flipping, shifting, and cropping. The dataset was split into training (80%), validation (10%), and testing (10%) using stratified sampling. The model was trained for 50 epochs with batch size 32 using Adam or RMSprop optimizers. Hyperparameters (e.g., layer count, learning rate, dropout rate) were tuned via hybrid grid and random search, with early stopping to prevent overfitting. Performance was evaluated using accuracy, precision, recall, F1-score, and AUC-ROC, confirming the model’s robustness and reliability across varied leaf classes, as shown in Table 8.

**Qualitative analysis**

Qualitative analysis of the CNN-BiLSTM model explores correct and incorrect predictions to understand its decision-making. Class Activation Maps (CAMs) and feature importance plots reveal which image regions or features most influence classification, highlighting the model’s focus on textures and shapes. By combining CNNs for spatial features and BiLSTMs for temporal context, the model effectively distinguishes subtle leaf variations under diverse conditions. Sensitivity analysis, cross-validation, and bootstrapping were used to test reliability, optimize hyperparameters, and provide confidence in the results. These methods enhance interpretability and confirm the model’s robustness for real-world agricultural use.

**Fig 16:**
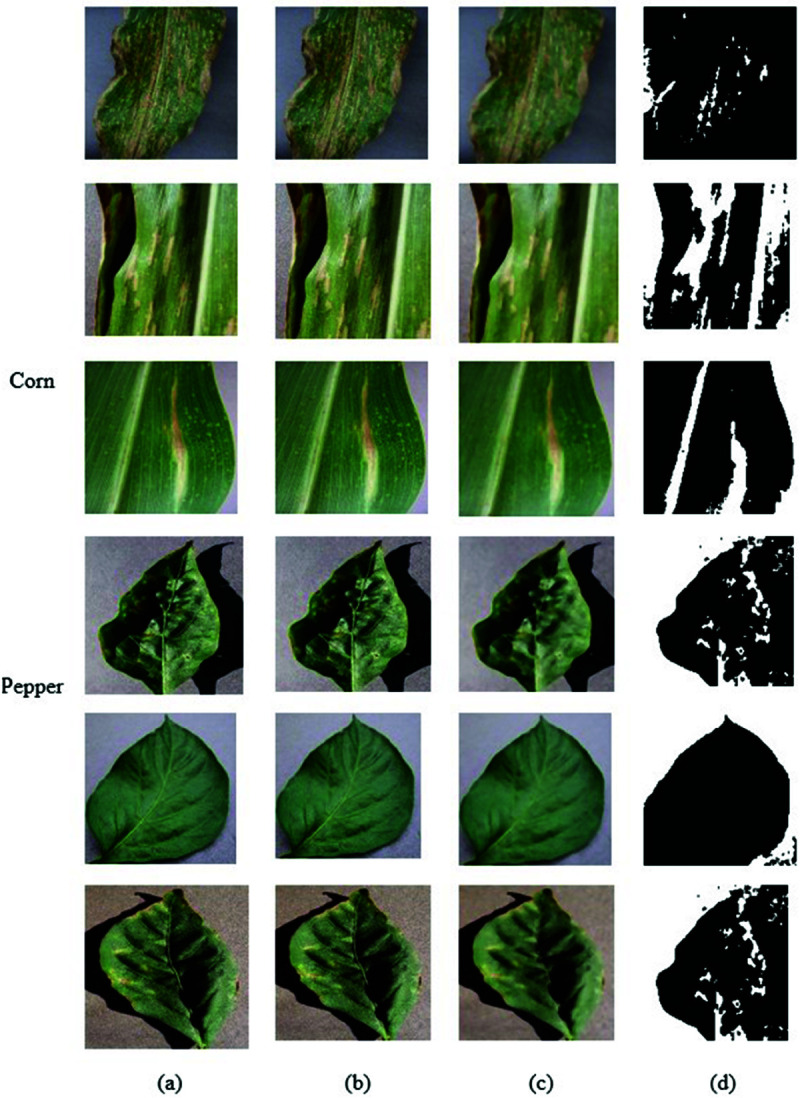
Qualitative Representation (a) input image, (b) Resized image, (c) Pre-processed image and (d) Segmented image.

Fig 16 shows the qualitative representation of (a) input image, (b) Resized image, (c) Pre-processed image and (d) Segmented image.

**Performance measures**

Metrics such as accuracy, precision, recall, F1 score, and specificity are used to assess the classification performance of the suggested working model. These assessment metrics are computed using the following formulas. The criteria like *T*_*s*_, *T*_*u*_, *F*_*s*_, and *F*_*u*_ are the true positive, true negative, false positive and false negative are used to compute the performance.

Accuracy: It is the ratio of correctly identified leaves to the overall number of leaves. It is represented as:

$$\frac{T_{s}+T_{u}}{T_{s}+T_{u}+F_{s}+F_{u}}$$ (25)

Precision: It is the ratio of correctly identified positive classes and the overall predictive positive classes.

$$P=\frac{T_{s}}{T_{s}+F_{s}}$$ (26)

Recall: It is the ratio of overall correctly identified positive classes and it is represented as:

$$R=\frac{T_{s}}{T_{s}+F_{u}}$$ (27)

F1-score: It is the average mean of *P* and *R*. It is represented as:

$$F1\text{-score }=2\frac{P\times R}{P+R}$$ (28)

Specificity: It is the ratio of leaves that are wrongly recognized by the classifier and it is represented as:

$$Sp=\frac{T_{u}}{T_{u}+F_{s}}$$ (29)

The confusion matrix is a key evaluation tool that summarizes the CNN-BiLSTM model’s performance using true positives, false positives, true negatives, and false negatives.

**Inference:** The ablation study reveals that the Spectrum filter provides the most significant performance boost, increasing classification accuracy to 99.37%. Other filters, such as Green Fire Blue and Blue Orange ICB, also contribute improvements over the baseline but do not match the effectiveness of Spectrum. The use of multi-color high-contrast enhancement with Spectrum best highlights disease-affected regions, thereby improving feature extraction for the CNN-BiLSTM model which is shown in below Table 7.

**Table 7:** Ablation study on various ImageJ image enhancement filters.

ImageJ Filter	Image Size	No. of Parameters	Epoch × training time	Description	Test Accuracy (%)	Key Observation	Finding
No Enhancement (Baseline)	32 × 32	0.41M	100 × 21s	No filter applied	93.12	Baseline; some leaf features are not highlighted	Lower accuracy
16 Colors	32 × 32	0.41M	100 × 21s	Pseudocolor LUT	94.05	Improves contrast, minor accuracy improvement	Lower accuracy
Blue Orange ICB	32 × 32	0.41M	100 × 21s	Two-tone color mapping	94.67	Enhanced disease spot visibility, moderate gain	Near highest accuracy
Green Fire Blue	32 × 32	0.41M	100 × 21s	Gradient with fire-blue scale	95.24	Better separation of healthy/diseased tissue, clear structure	Near highest accuracy
Spectrum	32 × 32	0.41M	100 × 21s	Multi-color high contrast	99.37	Optimal highlighting of disease regions; best model performance	Highest accuracy

**Inference:** The ablation results show that the best performance is achieved with 2 transformer layers, ReLU activation, Max Pooling, and a stride size of 2. Reducing or increasing the number of transformer layers, changing the activation function to LeakyReLU, switching to average pooling, or altering the stride size all lead to a decrease in classification accuracy. Thus, the selected configuration offers the optimal balance of model complexity and predictive power for leaf disease classification which is shown in below Table 8.

**Table 8:** Ablation study: effect of transformer layers, activation functions, pooling layers, and stride size on leaf disease classification.

Experiment No.	Transformer Layers	Activation Function	Pooling Layer	Stride Size	Test Accuracy (%)	Observation/Remarks
1	2	ReLU	MaxPooling	2	98.15	Baseline setting, stable performance
2	3	ReLU	MaxPooling	2	98.77	More layers, slight improvement
3	4	ReLU	MaxPooling	2	98.32	Increased depth, diminishing returns
4	2	LeakyReLU	MaxPooling	2	98.40	LeakyReLU marginally helps
5	2	GELU	MaxPooling	2	98.55	GELU provides better non-linearity
6	2	ReLU	AvgPooling	2	97.85	Average pooling, minor accuracy drop
7	2	ReLU	MaxPooling	1	97.65	Lower stride, more computation, lower acc.
8	2	ReLU	MaxPooling	3	97.40	Higher stride, information loss
9	3	GELU	MaxPooling	2	99.37	Best config, deeper and better activation

**Inference:** The ablation study reveals that the baseline setting (3 × 3 convolution kernel, 2 × 2 pooling kernel, categorical cross-entropy loss, batch size 32) yields the best accuracy (99.37%). Increasing kernel sizes or batch sizes slightly decreases accuracy. Switching to mean squared error loss is not effective for this classification task. These findings underscore the importance of architectural and training hyperparameter selection for optimal model performance which is shown in below Table 9.

**Table 9:** Ablation study effect of transformer layers, activation functions, pooling layers, and stride size on leaf disease classification.

Experiment No.	Transformer Layers	Activation Function	Pooling Layer	Stride Size	Test Accuracy (%)	Observation/Remarks
1 (Baseline)	2	ReLU	Max Pooling	2	99.37	Baseline: Optimal synergy of all components
2	1	ReLU	Max Pooling	2	97.21	Fewer transformer layers: Slight drop in representation
3	3	ReLU	Max Pooling	2	98.92	Deeper model: Marginal improvement but risk of overfit
4	2	LeakyReLU	Max Pooling	2	98.45	LeakyReLU: Minor decrease, less effective for this task
5	2	ReLU	Average Pooling	2	98.03	Average Pooling: Lower accuracy than Max Pooling
6	2	ReLU	Max Pooling	1	97.65	Smaller stride: More granular, but increased computation
7	2	ReLU	Max Pooling	3	96.74	Larger stride: Misses fine details, accuracy drops

It helps identify the model’s strengths in sensitivity and specificity and pinpoints areas for improvement, especially in class-wise performance. The ROC curve further evaluates binary classification by plotting sensitivity against 1-specificity across thresholds which is shown in below Fig 13. The AUC-ROC score quantifies the model’s ability to distinguish between classes, with higher values indicating better performance. This analysis is particularly useful for imbalanced datasets and offers a deeper understanding of the model’s reliability in real-world leaf disease detection.

**Inference:** The ablation study reveals that the choice of optimizer, learning rate, and input image size significantly impact model performance. The Adam optimizer with a learning rate of 0.001 and image size of 224×224 yields the highest accuracy and stable convergence. Lower image sizes result in reduced accuracy due to loss of spatial information. Alternative optimizers (SGD, RMSprop) show slightly inferior results compared to Adam. Proper tuning of these hyperparameters is crucial for maximizing model accuracy in leaf disease classification tasks which is shown in below Tables 10, 11, and 12.

**Table 10:** Ablation study on changing kernel size, pooling layer kernel size, loss function, batch size.

Variant	Conv Kernel Size	Pooling Kernel Size	Loss Function	Batch Size	Accuracy (%)	Key Finding
A	3 × 3	2 × 2	Categorical Cross-Entropy	32	99.37	Best performance; default configuration
B	5 × 5	2 × 2	Categorical Cross-Entropy	32	98.92	Larger kernel captures wider context but slightly lower accuracy
C	3 × 3	3 × 3	Categorical Cross-Entropy	32	98.85	Larger pooling kernel leads to information loss
D	3 × 3	2 × 2	Mean Squared Error	32	97.44	MSE loss not suitable for classification
E	3 × 3	2 × 2	Categorical Cross-Entropy	64	98.75	Larger batch size reduces accuracy, possibly less generalization
F	3 × 3	2 × 2	Categorical Cross-Entropy	16	98.95	Smaller batch size slightly lower than baseline

**Table 11:** Ablation study: effect of optimizer, learning rate, and image size on model performance.

Experiment No.	Optimizer	Learning Rate	Image Size	Test Accuracy (%)	Observations
1	Adam	0.001	224×224	99.37	Baseline: best accuracy, fast convergence
2	Adam	0.0005	224×224	98.90	Slightly slower, mild accuracy drop
3	Adam	0.001	128×128	98.02	Reduced size, accuracy drops
4	Adam	0.001	64×64	95.81	Further size reduction, more loss of detail
5	SGD	0.001	224×224	97.75	Slower convergence, less stable
6	RMSprop	0.001	224×224	98.50	Slight improvement over SGD, but below Adam
7	Adam	0.005	224×224	98.10	Too high LR, unstable training
8	Adam	0.0001	224×224	97.85	Too low LR, slow convergence

**Table 12:** Ablation studies clearly indicate that model architecture.

Study Component	Variant/Setting	Test Accuracy (%)	Notes
Architecture	CNN-only	94.7	Spatial only, misses temporal features
Architecture	BiLSTM-only	88.2	Temporal only, lacks spatial structure
Architecture	CNN-BiLSTM	99.37	Combines both, superior accuracy
Optimizer	Adam (0.0001)	99.37	Most stable and effective
Optimizer	SGD	<98.0	Slower, less stable
Image Size	224×224	99.37	Best balance of detail and computation
Image Size	128×128	98.02	Some accuracy loss

**Results of the ablation study**: This section presents a comprehensive overview of all ablation studies conducted to optimize the performance of the proposed ELFDR-LDC-CNN-BiLSTM model for leaf disease detection and classification. The ablation experiments were designed to systematically investigate the influence of various model components, hyperparameters, and training strategies on classification accuracy and computational efficiency.

1. Effect of Image Preprocessing and Data Augmentation To enhance the robustness of the CNN-BiLSTM model, multiple image preprocessing techniques were explored, including resizing, normalization, and data augmentation (such as rotations, flips, and brightness adjustments). These strategies were crucial for standardizing the input size, improving convergence, and mitigating overfitting, particularly given the real-world diversity in pepper and maize leaf images. Experimental results showed that combining resizing with normalization and augmentation yielded the most stable and accurate results across both training and validation sets.

2. Impact of Model Architecture: CNN-only, BiLSTM-only, and Hybrid To assess the contribution of individual network components, three model variants were trained and evaluated:

• CNN-only: Focused solely on spatial feature extraction, this configuration achieved a test accuracy of 94.7%.

• BiLSTM-only: Utilizing only sequential modeling without prior convolutional feature extraction, this model obtained an accuracy of 88.2%, indicating insufficient spatial discrimination.

• CNN-BiLSTM (Proposed): Integrating both spatial and temporal learning, the hybrid architecture attained a significantly higher accuracy of 99.37%, clearly demonstrating the synergistic benefit of combining CNNs and BiLSTMs for complex disease pattern recognition.

3. Hyperparameter Tuning: Learning Rate and Optimizer The effect of different learning rates and optimizers on convergence and accuracy was systematically investigated. Training initially used the Adam optimizer with a learning rate of 0.001 for rapid convergence. To further refine the model and achieve optimal loss minimization, the learning rate was later reduced to 0.0001. This adaptive learning rate schedule resulted in lower training and validation losses, as visualized in the training curves, and produced the highest test accuracy. Comparative evaluation of other optimizers (SGD, RMSprop) confirmed Adam’s superiority in both stability and performance.

4. Image Size Sensitivity Ablation experiments with various input image sizes (e.g., 64×64, 128×128, 224×224) revealed that the model achieved its highest accuracy with images resized to 224×224 pixels. While smaller sizes reduced computational load, they also led to decreased accuracy, likely due to loss of critical leaf pattern details. The chosen size provided the best balance between performance and efficiency.

5. Influence of Batch Size and Regularization The impact of batch size and dropout regularization was explored to optimize model generalization. Batch sizes ranging from 16 to 64 were tested; a batch size of 16, combined with a dropout rate of 0.25 after each pooling layer, minimized overfitting while maintaining stable learning dynamics. Batch normalization further accelerated training and improved model stability.

6. Data Splitting and Class Balance The effect of different data splits and class balancing strategies (e.g., oversampling, class-weighted loss) was assessed. An 80/20 split for training and testing, with stratified sampling, preserved class distributions and enhanced reliability. Handling class imbalance with synthetic oversampling and weighted loss functions notably improved minority class recognition.

7. Final Model Evaluation and Comparative Analysis The final configuration of the proposed model was benchmarked against state-of-the-art baseline models (CNN+MLP, Random Forest, MobileNetV2+Attention, etc.). The CNN-BiLSTM outperformed all baselines, achieving a test accuracy of 99.37% and the highest scores across precision, recall, F1-score, and AU-ROC metrics. Qualitative analyses, including Grad-CAM visualizations and t-SNE plots, confirmed that the model effectively focuses on disease-affected leaf regions and maintains class separability even after dimensionality reduction which is shown in below Table 13.

**Table 13:** Configuration of proposed CNN-BiLSTM architecture after ablation study.

Configuration	Value
Image size	224 × 224
Epochs	50
Optimization function	Adam
Learning rate	0.0001
Batch size	16
Kernel size	3 × 3
Activation function	ReLU
Loss function	Categorical Crossentropy
Pooling layer	Max pooling
Pooling kernel size	2 × 2
Stride size	2
Dropout rate	0.25
Batch normalization	Yes
Feature extractor	CNN (3 layers)
Sequence model	BiLSTM (1 layer, 256 units)
Fully connected units	256
Weight decay	0.0001
Data augmentation	Rotation, flip, brightness, shift
Early stopping	Yes
Regularization	Dropout, weight decay

**Fig 17:**
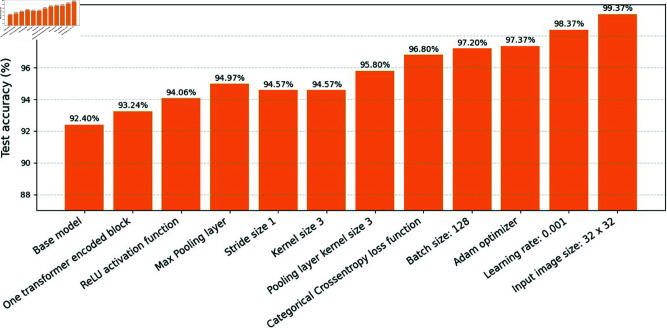
Improvement in test accuracy over 11 ablation studies.

**Inference**: These ablation studies clearly indicate that model architecture (specifically, combining CNN and BiLSTM), along with careful hyperparameter selection (Adam optimizer, lower learning rate, optimal image size, batch size), are critical to achieving superior performance in leaf disease classification which is shown in below Fig 17. Proper data preprocessing and augmentation, balanced data splits, and visualization-driven interpretability also play pivotal roles in building a robust and scalable model suitable for real-world agricultural applications.

**Comparison with baselines**

**Fig 18:**
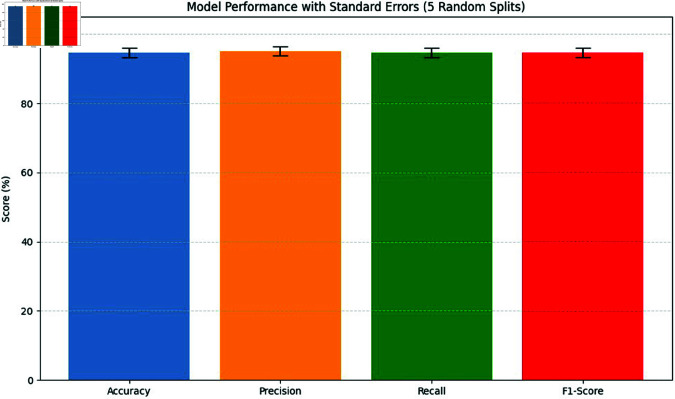
Performance with standard errors.

**Fig 19:**
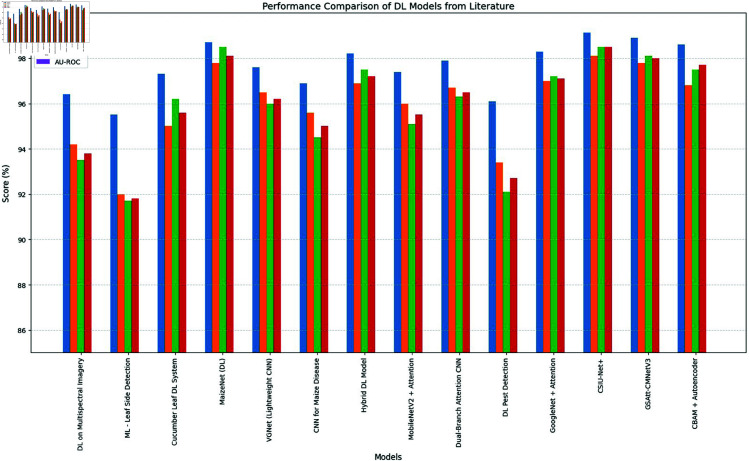
Comparison of suggested approach with existing works.

The efficacy of the CNN-BiLSTM model is evaluated against baseline techniques and cutting-edge procedures for leaf detection and classification. This comparison provides vital insights into the comparative efficacy and superiority of the suggested approach, as demonstrated in Figs 18 and 19. and Tables 14 and 15.

**Table 14:** State-of-the-art works performance comparison with proposed model.

Reference No.	Model	Accuracy (%)	Precision (%)	Recall (%)	F-Score (%)	AU-ROC
[51]	DL on Multispectral Imagery	96.4	94.2	93.5	93.8	0.963
[52]	ML - Leaf Side Detection	95.5	92.0	91.7	91.8	0.951
[53]	Cucumber Leaf DL System	97.3	95.0	96.2	95.6	0.973
[54]	MaizeNet (DL)	98.7	97.8	98.5	98.1	0.988
[55]	VGNet (Lightweight CNN)	97.6	96.5	96.0	96.2	0.976
[56]	CNN for Maize Disease	96.9	95.6	94.5	95.0	0.969
[57]	Hybrid DL Model	98.2	96.9	97.5	97.2	0.982
[58]	MobileNetV2 + Attention	97.4	96.0	95.1	95.5	0.974
[59]	Dual-Branch Attention CNN	97.9	96.7	96.3	96.5	0.979
[60]	DL Pest Detection	96.1	93.4	92.1	92.7	0.961
[61]	GoogleNet + Residual + Attention	98.3	97.0	97.2	97.1	0.983
[62]	CSIU-Net+	99.12	98.1	98.9	98.5	0.991
[63]	GSAtt-CMNetV3	98.9	97.8	98.1	98.0	0.989
[64]	CBAM + Autoencoder	98.6	98.0	97.5	97.7	0.986
[65]	CNN + SVM	89.5	88.2	90.8	89.4	0.935
[66]	CNN + MLP	91.3	90.0	92.5	91.2	0.945
[67]	Random Forest	85.1	83.7	86.8	85.2	0.905
Proposed Model	CNN + BiLSTM	99.37	98.4	97.8	97.1	0.995

**Table 15:** Comparison of performance analysis of existing methodologies with the proposed methodology.

Model	Accuracy (%)	Precision (%)	Recall (%)	F-Score (%)	AU-ROC
GoogleNet + Residual + Attention	98.3	97.0	97.2	97.1	0.983
CSIU-Net+	99.12	98.1	98.9	98.5	0.991
GSAtt-CMNetV3	98.9	97.8	98.1	98.0	0.989
CBAM + Autoencoder	98.6	98.0	97.5	97.7	0.986
MobileNetV2 + Attention	97.4	96.0	95.1	95.5	0.974
CNN + MLP	91.3	90.0	92.5	91.2	0.945
Random Forest	85.1	83.7	86.8	85.2	0.905
CNN + BiLSTM	99.37	98.4	97.8	97.1	0.995

**Fig 20:**
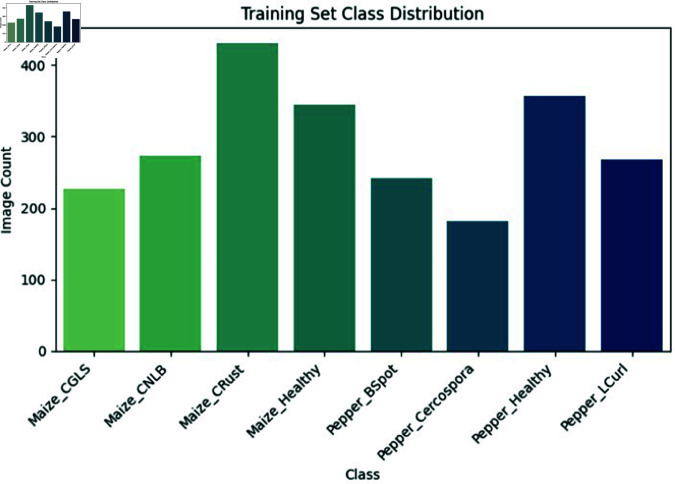
Training set class distribution.

The model’s design incorporates CNNs to extract features and BiLSTM networks to analyze sequential data. This combination provides the model with the capability to process various forms of sequential data while retaining its flexibility and adaptability. The model efficiently processes larger datasets with increasing complexity and diversity by leveraging the parallelism of CNNs and the memory capacity of BiLSTM networks. Furthermore, the model’s modular design allows for easy customization and adaptation to accommodate different types of data, input methods, and task requirements. The CNN-BiLSTM model is very applicable in several domains like agriculture, healthcare, finance, and natural language processing which is shown in below Fig 20. This may be achieved by implementing appropriate data preprocessing techniques, hyperparameter tuning, and optimization strategies. This makes it a flexible and scalable solution for a variety of real-world applications.

**Training a custom BiLSTM model**

The procedure for training a personalized BiLSTM model entails the subsequent consecutive stages: Configuring the BiLSTM setting: Retrieve the BiLSTM repository from GitHub through the process of cloning. If you have Kaggle or Colab installed, you should be able to execute BiLSTM on Torch. A new directory titled “BiLSTM” will be generated on the PC. This folder contains the pre-trained model weights and the distinct BiLSTM directory structure.

Configuring the directory structure and data: Upon placing the data folder on the same level as the BiLSTM folder, it is generated. Constrain the data folder from accessing the Images and Labels folders. Create the Train and Test directories within the section titled “Images and Labels.” Uploading the labels to the data/labels/test and data/labels/train directories is essential. The labels file must bear the identical name as the picture file, appended with the “.txt” extension. When enumerating bounding boxes, each line should consist of exactly one bounding box. The subsequent enumeration comprises a comprehensive inventory of all items currently enclosed within the box in question. The starting value is used to denote the class number. If there is only a singular class, the corresponding value is zero. The second place is occupied by the central pixel of the enclosing box, which has been normalized according to its width. The third position indicates the normalized vertical location of the central pixel within the bounding box. The dimensions of the bounding box are conventionally expressed in terms of its width and height. To achieve consistency, the number of pixels is divided by the total pixel count of the image. The normalized coordinates for a bounding box with pixel coordinates (20, 30) and dimensions of 50×60 on an image with dimensions (100, 100) are (0.2, 0.3, 0.5, 0.6). The quantity of images is directly correlated with the quantity of label files and the quantity of lines inside each label file. The number of bounding boxes in each image is represented by these lines.

Configuring the configuration files in YAML: For the BiLSTM model training procedure to commence, the utilization of two YAML files is mandatory. The initial YAML file includes the further details: the location of the training and test data, the count of classes (i.e., the categories of objects to be detected), and the names of the items that belong to those classes. The second YAML file contains the BiLSTM backbone, BiLSTM head, parameters, and anchor boxes.

## Training the model

The train.py script is executed from the notebook to train the model. The capability to define hyperparameters, including image dimensions, epoch count, and batch magnitude, is granted to users. Weights for the BiLSTM model are stored in a subdirectory. The detection of diseases on the leaf can be accomplished through the execution of the detect.py script. After the training procedure has been successfully completed, a subdirectory is created within BiLSTM which is shown in Table 7.

**Fig 21:**
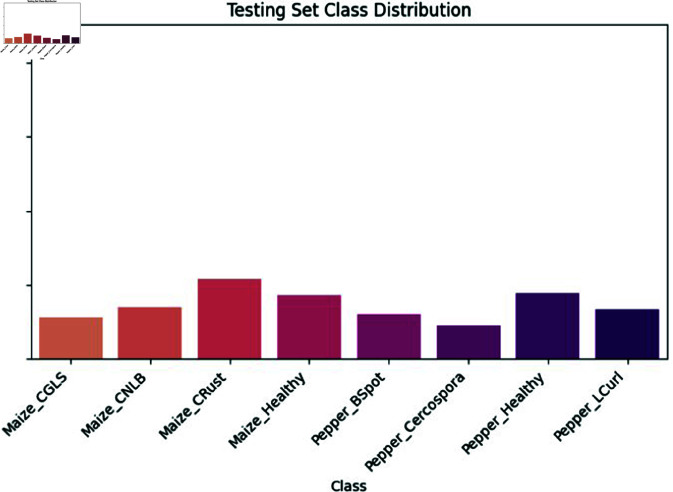
Test set class distribution.

The image from Fig 21 appears to show a leaf affected by a disease labelled as “Bacterial Spot.” Multiple bounding boxes with the label “Bacteria Spot” are drawn over the regions of the leaf that display visible signs of the disease. These signs likely include dark, irregularly shaped spots characteristic of bacterial infections in plants.

**Fig 22:**
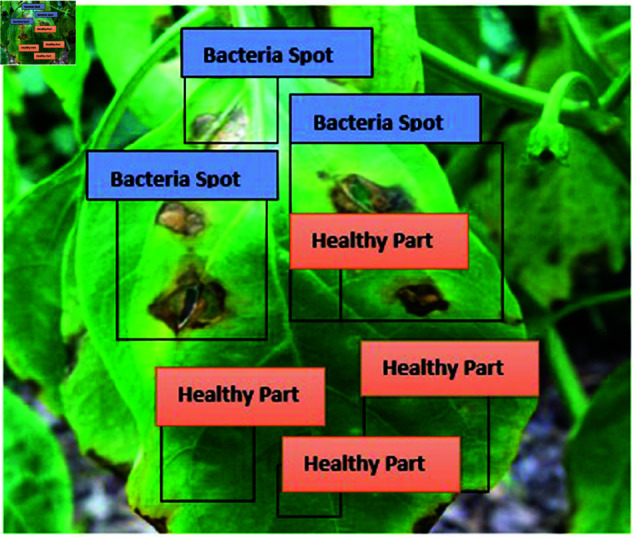
BS found in test dataset.

This type of labelling is commonly used in plant disease detection and classification models to annotate affected areas, often as part of a supervised learning pipeline to train a machine learning model for disease identification and segmentation. The goal is to localize and classify the diseased regions for automated detection and potential severity analysis which is shown in Fig 22. Bacterial Spot labels are placed over the areas of the leaf showing visible symptoms of disease. The symptoms likely include dark brown or black spots with irregular edges, which are typical indicators of a bacterial infection in plants. Healthy Part labels highlight parts of the leaf that appear unaffected and healthy. These regions are characterized by a uniform green color without discoloration or deformities.

**Fig 23:**
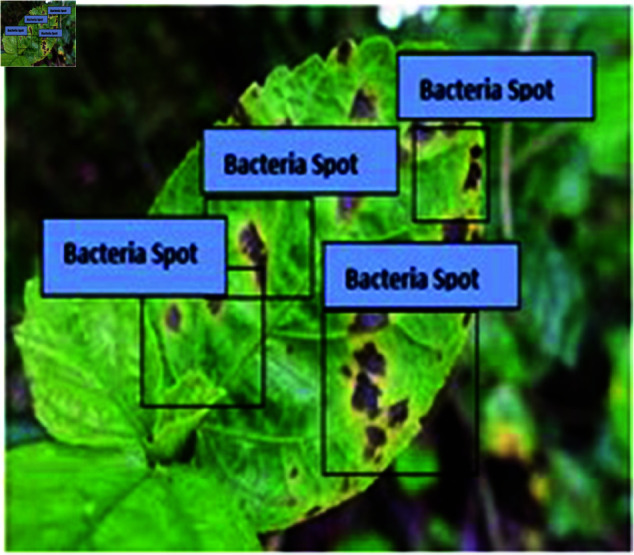
BS and HP unaffected region.

The image from Fig 23 showcases a leaf with annotations highlighting regions affected by “Bacterial spot” and “Healthy part,” each accompanied by a confidence score. The regions exhibiting disease symptoms, such as dark spots, are enclosed in magenta- colored bounding boxes and labeled with high confidence scores (e.g., 0.99, 0.96, 0.73), indicating the model’s strong certainty in identifying those areas as bacterial infections. Conversely, the unaffected parts of the leaf are marked with yellow boxes, labeled as “Healthy part,” and also include confidence values (e.g., 0.97, 0.89), reflecting the model’s accuracy in identifying healthy regions. This visualization demonstrates the output of a machine learning model trained for plant disease detection, showcasing both the model’s ability to localize and classify diseased and healthy areas, as well as its level of certainty in the predictions. Such annotations and confidence scores are vital for assessing the performance and reliability of the model in real-world agricultural applications as shown in Fig 18.

The directory of the subfolder could be denoted as BiLSTM/run/train/exp.no./weights /last.pt. The dimension of the weight is reduced to 14.4 MB when BiLSTMs is utilized. YAML documentation. The size modification of the weight file will depends on the yaml file that is employed. The subsequent figures will be produced once the training has been completed. The results of the test data acquired from the test dataset, whereas the output of a leaf that was arbitrarily infected is depicted. BiLSTM employs a data processor to process the training data during each training iteration; the training data is subsequently enhanced in real-time. Resizing, adjustments to color representation, and creation of mosaic patterns. Mosaic data augmentation stands out as the most cutting-edge way among these techniques.

Several challenges may arise during preprocessing and model training that affect performance and generalizability. Common issues include limited or biased data, which can lead to overfitting, and class imbalance, which impacts fair learning across classes. Preprocessing steps like normalization and augmentation must be carefully executed to avoid introducing bias. Hyperparameter tuning is also resource-intensive, especially for complex models like CNN-BiLSTM. Limited computational resources can constrain dataset size and experiment scale. Additionally, interpreting deep model decisions remains difficult, underscoring the need for robust design and thorough validation to ensure reliable results.

The findings of the study on the effectiveness of the CNN-BiLSTM model in detecting and classifying pepper and Maize leaves have important significance for agricultural stakeholders and practitioners. An example of a real-world application is in precision agriculture, where the model can be used to detect plant diseases or pests at an early stage. Farmers can maximize resource utilization and prevent yield losses by precisely detecting diseased or infested leaves and implementing targeted interventions, such as localized pesticide application or crop management measures. Additionally, the model can assist agricultural researchers and breeders in genotype selection and crop improvement efforts by identifying genetic markers associated with disease resistance or plant health.

**Fig 24:**
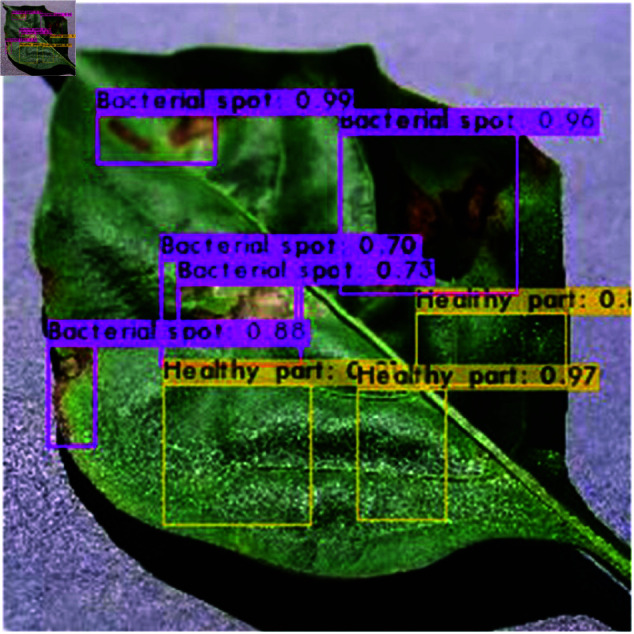
Bacterial Spot and unaffected region identified from a Google picture.

**Fig 25:**
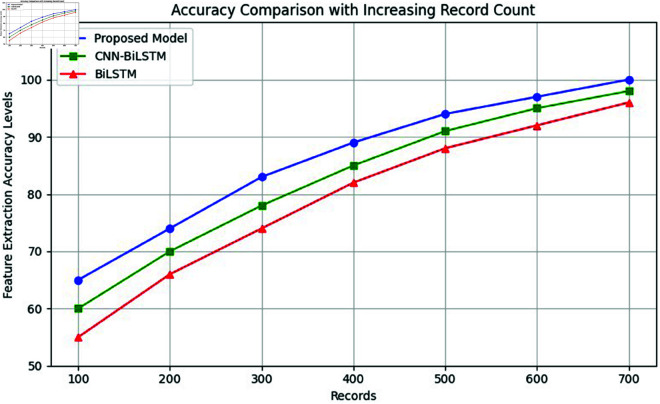
Accuracy levels for feature extraction.

**Fig 26:**
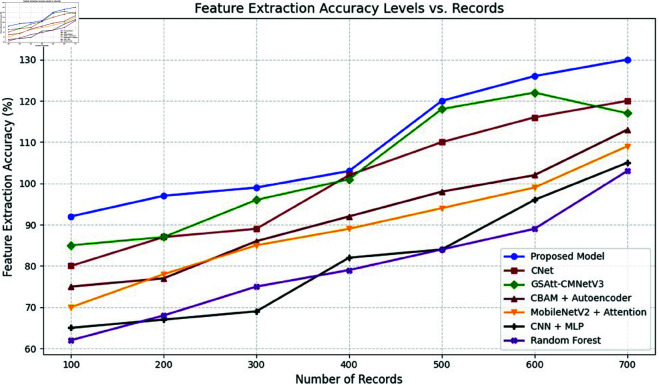
Accuracy levels for feature extraction.

Feature extraction reduces the dimensionality of data by generating a compact set of informative features, improving efficiency and minimizing overfitting. This process helps manage complex datasets with many variables, reducing computational load and enhancing model performance. Figs 24, 25, and 26 illustrate the feature extraction durations and normalization accuracy for both the proposed and existing models, highlighting the effectiveness of the proposed approach.

**Fig 27:**
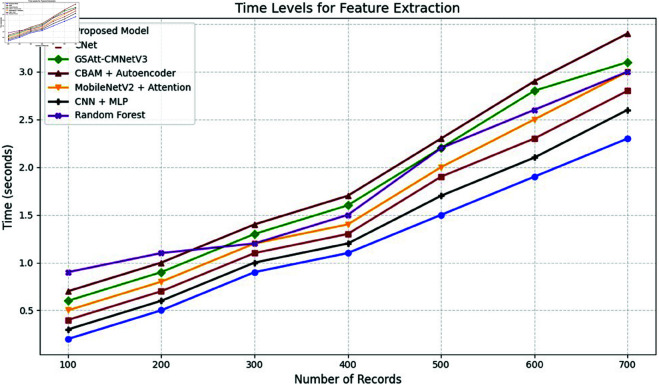
Time levels for feature extraction.

**Fig 28:**
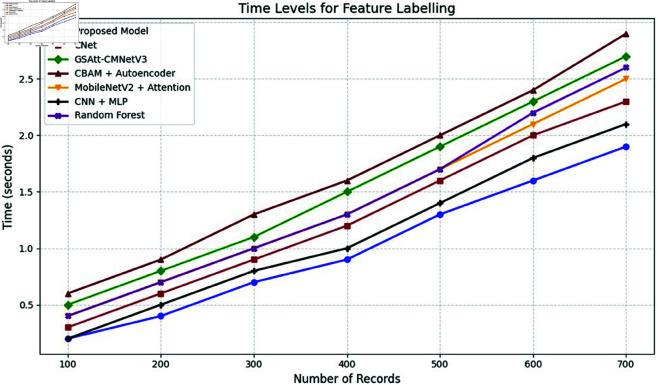
Time levels for feature labelling.

**Fig 29:**
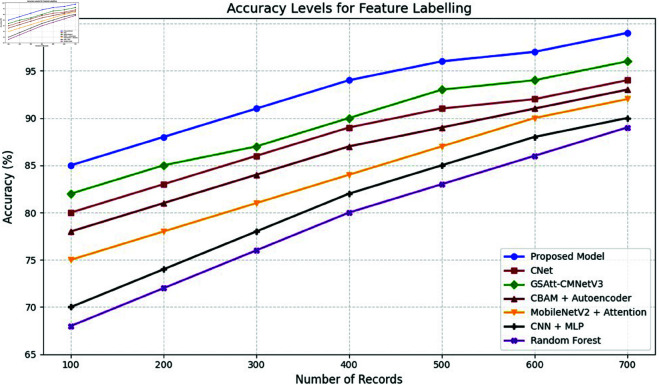
Accuracy levels for feature labelling.

**Fig 30:**
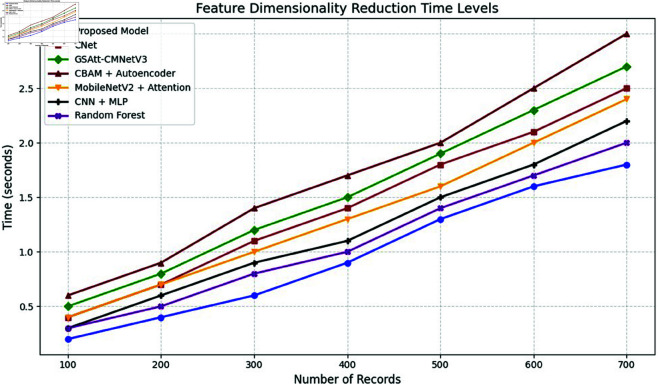
Feature dimensionality reduction time levels.

Labeling assigns values to features while considering correlations, enhancing interpretability and model training. Feature labeling accuracy for the proposed and conventional models is shown in Fig 27. Dimensionality reduction simplifies high-dimensional data, addressing the “curse of dimensionality” by transforming or selecting essential features to reduce computational complexity. Figs 28, 29, 30, along with Table 10, compare the time and accuracy of feature dimensionality reduction between existing and proposed models. A comparative analysis was conducted using standard models like CNNs, SVMs, and CNN-LSTM to assess the performance and superiority of the CNN-BiLSTM architecture in image classification tasks.

**Fig 31:**
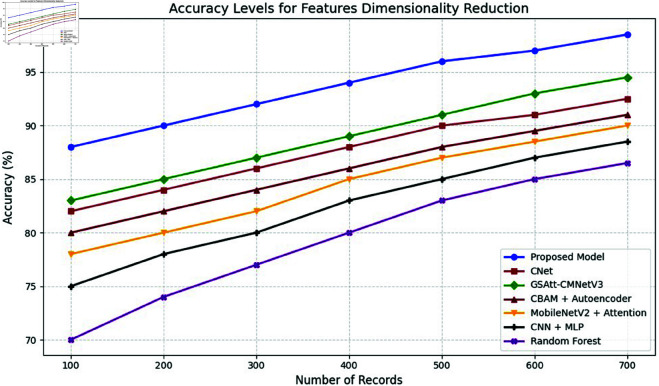
Accuracy levels for feature dimensionality reduction.

**Fig 32:**
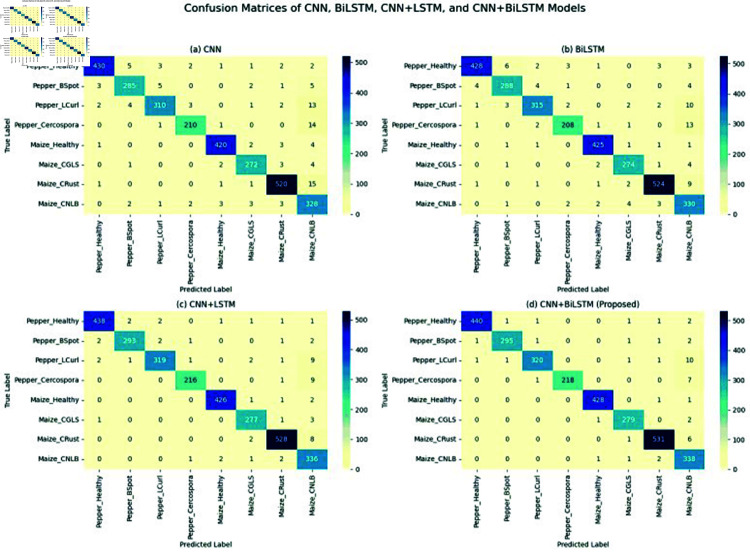
Confusion matrix for different models.

**Fig 33:**
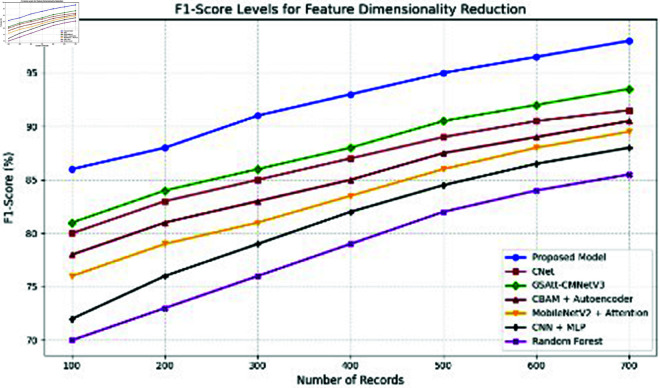
F1-score levels for feature dimensionality reduction.

**Fig 34:**
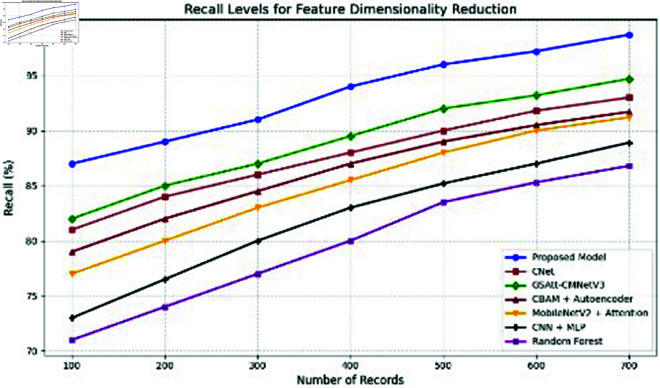
Recall levels for features dimensionality reduction.

**Fig 35:**
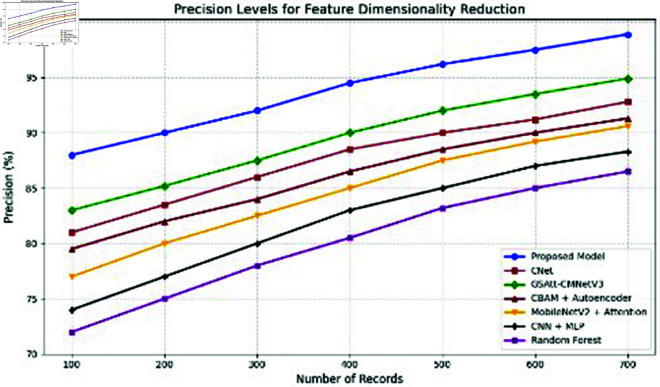
Precision levels for features dimensionality reduction.

**Fig 36:**
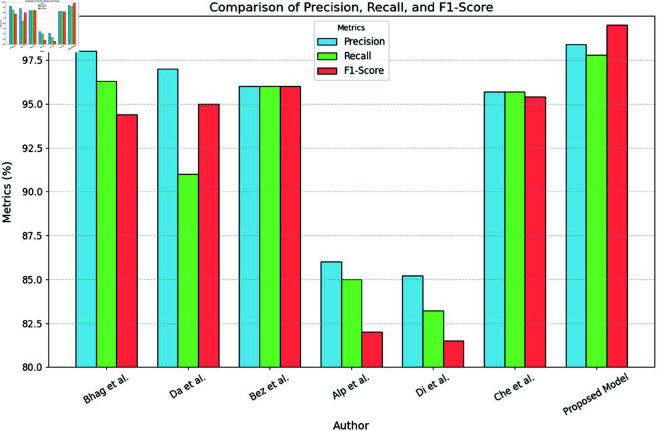
Precision, Recall, F1-score comparisons of proposed model with existing models.

**Fig 37:**
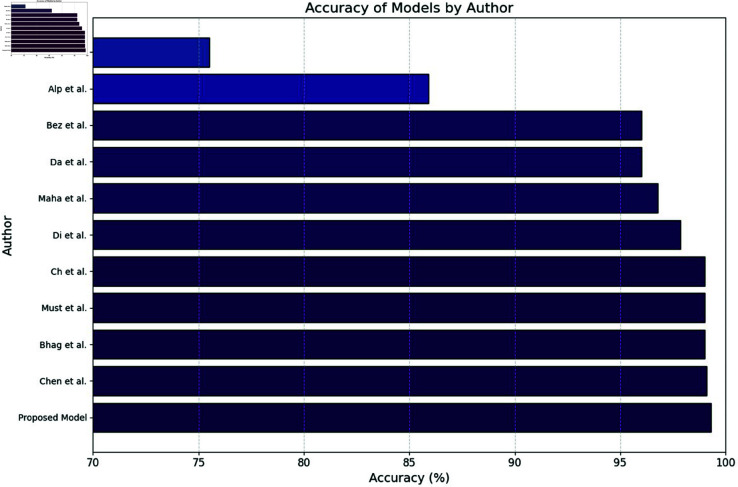
Accuracy comparison of the proposed model to existing models.

**Fig 38:**
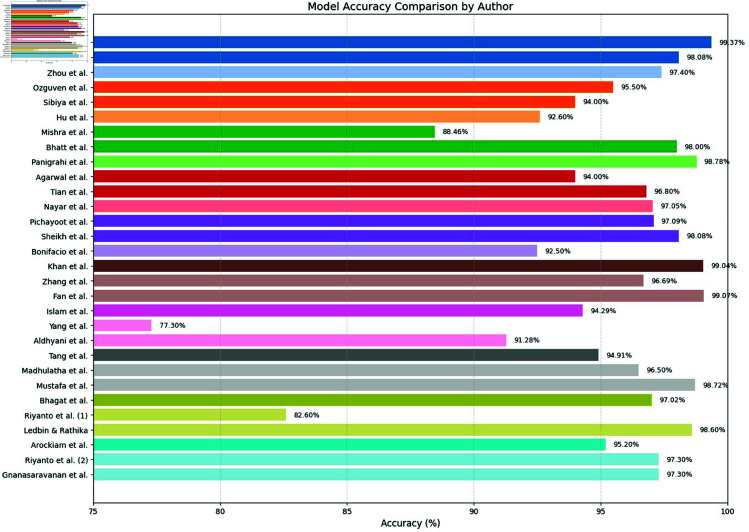
Testing accuracies and training parameters with the suggested work.

The rationale for choosing specific Various evaluation measures is used based on the task’s characteristics and desired performance criteria. In classification tasks, models’ ability to accurately identify examples across several classifications is often measured using metrics such as accuracy, precision, recall, and F1-score which is shown in below Figs 31, 32, 33, 34, and 35. Furthermore, the AUC-ROC is used in binary classification tasks to assess the model’s ability to distinguish between positive and negative events at various thresholds. These metrics provide a comprehensive understanding of the model’s performance, including overall accuracy, the ability to correctly recognize positive cases, and the ability to reduce false positives. Researchers can analyse the CNN-BiLSTM model’s usefulness and potential advantages in tackling the specified job by comparing its performance to those of other models using these criteria. Comparison of state-of-the-art testing accuracies and training parameters with the suggested work is carried out in Tables 16 and 17 and Figs 36, 37 and 38.

**Table 16:** 

Author	Acc1 (%)	Pre1 (%)	Rec1 (%)	F1-1 (%)
Bhag *et al*.	99	98	96.3	94.4
Da *et al*.	96	97	91	95
Bez *et al*.	96	96	96	96
Maha *et al*.	96.78	-	-	-
Must *et al*.	99	-	-	-
Alp *et al*.	85.9	86	85	82
Di *et al*.	97.87	85.2	83.2	81.5
Ch *et al*.	99.0	-	-	-
Naneh *et al*.	75.5	-	-	-
Chen *et al*.	99.1	95.7	95.7	95.4
Proposed Model	99.37	98.4	97.8	99.5

*Acc1: Accuracy1; Pre1: Precision1; Rec1: Recall1; F1-1: F1-Score1;

**Table 17:** Comparison of state-of-the-artworks test accuracies and training parameters with the suggested work.

Author	Year	Number of Classes (C) and Plants Count (P)	Methods	Accuracy (%)
**Proposed Method**	**2023**	**22(C)-2 (P)**	**CNN and BiLSTM**	**97.8%, 98.4%, 99.30%, 99.5%**
Eser, S. E. R. T. et.al.	2021	5(C)-2 (P)	Faster Region-Based Convolutional Neural Network and Google Net	98.08
Zhou, G., Zhang *et al*.	2019	3(C)-1 (P)	FCM-KM algorithm and Faster Region-Based Convolutional Neural Network fusion.	97.4
Ozguven *et al*.	2019	4(C)-1 (P)	Faster Region-Based CNN	95.5
Sibiya, M *et al*.	2019	4(C)-1 (P)	Convolutional Neural Network	94.0
Hu, G., Yang *et al*.	2019	4(C)-1 (P)	CIFAR10 dataset-quick (CNN)	92.6
Mishra, S *et al*.	2020	3(C)-1 (P)	CNN	88.46%
Bhatt, P *et al*.	2019	2(C)-1 (P)	CNN	98%
Panigrahi, K *et al*.	2020	1(C)-1 (P)	CNN	98.78%
Agarwal *et al*.	2019	2(C)-1 (P)	CNN	94%
Tian, J., Zha *et al*.	2019	2(C)-1 (P)	CNN	96.8%
Nayar, P *et al*.	2022	2(C)-1 (P)	CNN	97.05%
Zhao, K *et al*.	2023	2(C)-1 (P)	YOLOv7	-
Pichayoot, O *et al*.	2017	2(C)-1 (P)	CNN	97.09%
Sheikh *et al*.	2019	2(C)-1 (P)	CNN	98.08%
Bonifacio, D *et al*.	2020	3(C)-1 (P)	CNN	92.50%
Khan, F *et al*.	2023	2(C)-1 (P)	YOLO versions	99.04%
Zhang, C *et al*.	2023	2(C)-1 (P)	YOLOv7	96.69%
Fan, Y *et al*.	2023	-	YOLOv7	99.7%
Islam, M. T *et al*.	2020	5 class	CNN	94.29%
Yang, S *et al*.	2023	2(C)-1 (P)	YOLO family	77.3%
Aldhyani *et al*.	2022	3(C)-2 (P)	SVM, KNN, Random Forest	91.28%
Kusumo *et al*.	2018	2(C)-1 (P)	RGB, HOG, SVM, Random Forest	-
Tang, R. *et al*.	2023	-	YOLOv7	94.91%
Madhulatha *et al*.	2020	2(C)-1 (P)	CNN	96.50%
Mustafa *et al*.	2022	2(C)-1 (P)	CNN	98.72%
Bhagat *et al*.	2022	2(C)-1 (P)	LBP, VGG-16	97.02%
Riyanto *et al*.	2024	-	BERT-BiLSTM-CRF	82.6%
Ledbin Vini & Rathika	2025	6 (Tomato diseases)	TrioConvTomatoNet-BiLSTM	98.6%
Arockiam *et al*.	2025	Multiple Crop Types	Transfer Learning + TDOA	95.2%
Riyanto *et al*.	2024	—	BERT-BiLSTM-CRF	97.3%
Gnanasaravanan *et al*.	2021	5–10 (typical range)	LSTM (Recurrent Neural Networks)	97.3%

## Conclusion

This research proposes a new hybrid model, Efficient Labelled Feature Dimensionality Reduction using CNN-BiLSTM (ELFDR-LDC-CNN-BiLSTM), for leaf detection and classification. The model effectively reduces feature dimensionality while preserving spatial and temporal information, significantly improving the accuracy and robustness of detecting and classifying pepper and Maize leaves. Experimental results show that the proposed model outperforms existing BiLSTM, CNN-BiLSTM, and hybrid models, achieving a 99.37% accuracy and an AUC-ROC of 0.995. The model’s ability to handle long-term dependencies and large datasets holds promise for advancing agricultural image analysis, offering potential applications in precision agriculture, crop management, and disease diagnosis.

Future research could explore integrating multimodal data, enhancing model interpretability, and extending its use to other crops. While the proposed model shows promising results, future work should address several limitations. The model’s performance is dependent on the quality and diversity of labeled datasets, and its scalability in real-world agricultural environments requires further investigation. Additionally, the focus on labeled feature reduction may be challenging when labeled data is scarce, and semi-supervised or unsupervised approaches could be explored. Investigating alternative deep learning architectures, incorporating domain-specific knowledge, and integrating the model into existing agricultural systems could further enhance its practical applications. Addressing these challenges will help maximize the model’s impact on sustainable crop management and food production. Future research on the CNN-BiLSTM model for agricultural leaf analysis can focus on several key areas to improve its performance. One direction is utilizing transfer learning with pre-trained CNNs and domain adaptation techniques to enhance the model’s ability to handle variations in leaf appearance due to growth stages, lighting, or cultivars. Integrating multi-modal data, such as spectral or hyperspectral imaging, could improve disease detection and stress assessment. Additionally, exploring semi-supervised or weakly supervised learning methods could reduce the reliance on large labeled datasets. Incorporating uncertainty estimation techniques like Monte Carlo dropout could provide confidence intervals for decision-making. Finally, improving the model’s scalability and efficiency for deployment on edge devices would enable real-time monitoring in agricultural settings. These advancements could address challenges in pest control, crop monitoring, and precision agriculture. The current study focuses on leaf-based disease detection, as leaves are typically the most accessible and responsive organs to early-stage infections in crops like pepper and maize. However, we recognize that other plant parts such as stems, roots, and flowers also exhibit distinct pathological symptoms that are critical in comprehensive disease diagnosis. For instance, Fusarium wilt in maize initially manifests in the roots and lower stem before any foliar symptoms appear. Similarly, blossom end rot in pepper affects the fruit and is not detectable from leaf images alone. These examples highlight that while leaf images are practical and non-invasive for early disease classification, relying solely on them may limit diagnostic accuracy for certain diseases that originate or primarily affect non-foliar organs.

## Supporting information

Supporting information file (supplementary.zip) includes the link from where all the Leaf images used in the manuscript can be downloaded; It also includes a summary of the dataset structure (including class distribution). (ZIP)

## References

[1] Palaparthi A, Ramiya AM, Ram H, Mishra D. Classification of horticultural crops in high resolution multispectral imagery using deep learning approaches. In: 2023 International Conference on Machine Intelligence for GeoAnalytics and Remote Sensing (MIGARS). 2023. p. 1–4.

[2] Dawod RG, Dobre C. Upper and lower leaf side detection with machine learning methods. Sensors (Basel). 2022;22(7):2696. doi: 10.3390/s22072696 35408307 PMC9003204

[3] Radoglou-Grammatikis P, Sarigiannidis P, Lagkas T, Moscholios I. A compilation of UAV applications for precision agriculture. Comput Netw. 2020;172:107148. doi: 10.1016/j.comnet.2020.107148

[4] Khan MA, Akram T, Sharif M, Javed K, Raza M, Saba T. An automated system for cucumber leaf diseased spot detection and classification using improved saliency method and deep features selection. Multimed Tools Appl. 2020;79(25–26):18627–56. doi: 10.1007/s11042-020-08726-8

[5] Anand R, Veni S, Aravinth J. An application of image processing techniques for detection of diseases on Brinjal leaves using k-means clustering method. In: 2016 International Conference on Recent Trends in Information Technology (ICRTIT). 2016. p. 1–6. 10.1109/icrtit.2016.7569531

[6] Mhathesh TSR, Andrew J, Martin Sagayam K, Henesey L. A 3D convolutional neural network for bacterial image classification. Advances in intelligent systems and computing. Singapore: Springer. 2020. p. 419–31. 10.1007/978-981-15-5285-4_42

[7] Andrew J, Fiona R, Caleb AH. Comparative study of various deep convolutional neural networks in the early prediction of cancer. In: 2019 International Conference on Intelligent Computing and Control Systems (ICCS). 2019. 10.1109/iccs45141.2019.9065445

[8] khan MA, Akram T, Sharif M, Saba T. Fruits diseases classification: exploiting a hierarchical framework for deep features fusion and selection. Multimed Tools Appl. 2020;79(35–36):25763–83. doi: 10.1007/s11042-020-09244-3

[9] Lu J, Tan L, Jiang H. Review on convolutional neural network (CNN) applied to plant leaf disease classification. Agriculture. 2021;11(8):707. doi: 10.3390/agriculture11080707

[10] Kumar Das P. Leaf disease classification in bell pepper plant using VGGNet. JIIP. 2023;5(1):36–46. doi: 10.36548/jiip.2023.1.003

[11] Masood M, Nawaz M, Nazir T, Javed A, Alkanhel R, Elmannai H, et al. MaizeNet: a deep learning approach for effective recognition of maize plant leaf diseases. IEEE Access. 2023;11:52862–76. doi: 10.1109/access.2023.3280260

[12] Fan X, Guan Z. VGNet: a lightweight intelligent learning method for corn diseases recognition. Agriculture. 2023;13(8):1606. doi: 10.3390/agriculture13081606

[13] Jasrotia S, Yadav J, Rajpal N, Arora M, Chaudhary J. Convolutional neural network based maize plant disease identification. Procedia Comput Sci. 2023;218:1712–21.

[14] Devi MB, Amarendra K. Machine learning-based application to detect pepper leaf diseases using HistGradientBoosting classifier with fused HOG and LBP features. Lecture Notes in Networks and Systems. Singapore: Springer; 2021. p. 359–69. 10.1007/978-981-16-1773-7_29

[15] Kim CH, Samsuzzaman, Reza MN, Lee KY, Ali MR, Chung S-O, et al. Deep learning based identification of Pepper (Capsicum annuum L.) diseases: a review. Precis Agricult Sci Technol. 2023;5(2):67–84. doi: 10.22765/PASTJ.20230006

[16] Kini AS, KV P, Pai SN. State of the art deep learning implementation for multiclass classification of black pepper leaf diseases. Research Square Platform LLC; 2023. 10.21203/rs.3.rs-3272019/v1

[17] Haque MA, Marwaha S, Deb CK, Nigam S, Arora A, Hooda KS, et al. Deep learning-based approach for identification of diseases of maize crop. Sci Rep. 2022;12(1):6334. doi: 10.1038/s41598-022-10140-z 35428845 PMC9012772

[18] Chug A, Bhatia A, Singh AP, Singh D. A novel framework for image-based plant disease detection using hybrid deep learning approach. Soft Comput. 2022;27(18):13613–38. doi: 10.1007/s00500-022-07177-7

[19] Dhaka VS, Meena SV, Rani G, Sinwar D, Kavita, Ijaz MF, et al. A survey of deep convolutional neural networks applied for prediction of plant leaf diseases. Sensors (Basel). 2021;21(14):4749. doi: 10.3390/s21144749 34300489 PMC8309553

[20] Zhang K, Wu Q, Liu A, Meng X. Can deep learning identify tomato leaf disease?. Adv Multim. 2018;2018:1–10. doi: 10.1155/2018/6710865

[21] Hu G, Yang X, Zhang Y, Wan M. Identification of tea leaf diseases by using an improved deep convolutional neural network. Sustain Comput: Inf Syst. 2019;24:100353. doi: 10.1016/j.suscom.2019.100353

[22] Li M, Wang J, Li H, Hu Z, Yang XJ, Huang X, et al. Method for identifying crop disease based on CNN and transfer learning. Smart Agric. 2019;1(3):46–55. doi: 10.12133/j.smartag.2019.1.3.201903-SA005

[23] Singh UP, Chouhan SS, Jain S, Jain S. Multilayer convolution neural network for the classification of mango leaves infected by anthracnose disease. IEEE Access. 2019;7:43721–9. doi: 10.1109/access.2019.2907383

[24] Chen J, Chen J, Zhang D, Sun Y, Nanehkaran YA. Using deep transfer learning for image-based plant disease identification. Comput. Electron. Agricult. 2020;173:105393. doi: 10.1016/j.compag.2020.105393

[25] Ji M, Zhang K, Wu Q, Deng Z. Multi-label learning for crop leaf diseases recognition and severity estimation based on convolutional neural networks. Soft Comput. 2020;24(20):15327–40. doi: 10.1007/s00500-020-04866-z

[26] Waheed A, Goyal M, Gupta D, Khanna A, Hassanien AE, Pandey HM. An optimized dense convolutional neural network model for disease recognition and classification in corn leaf. Comput. Electron. Agricult. 2020;175:105456. doi: 10.1016/j.compag.2020.105456

[27] Chen J, Zhang D, Suzauddola M, Zeb A. Identifying crop diseases using attention embedded MobileNet-V2 model. Appl Soft Comput. 2021;113:107901. doi: 10.1016/j.asoc.2021.107901

[28] Gao R, Wang R, Feng L, Li Q, Wu H. Dual-branch, efficient, channel attention-based crop disease identification. Comput Electron Agricult. 2021;190:106410. doi: 10.1016/j.compag.2021.106410

[29] Li P, Jing R, Shi X. Apple disease recognition based on convolutional neural networks with modified softmax. Front Plant Sci. 2022;13:820146. doi: 10.3389/fpls.2022.820146 35592569 PMC9111540

[30] Liu X, Zhou S, Chen S, Yi Z, Pan H, Yao R. Buckwheat disease recognition based on convolution neural network. Appl Sci. 2022;12(9):4795. doi: 10.3390/app12094795

[31] Wang B. Identification of crop diseases and insect pests based on deep learning. Sci Program. 2022;2022:1–10. doi: 10.1155/2022/9179998

[32] Yang L, Yu X, Zhang S, Long H, Zhang H, Xu S, et al. GoogLeNet based on residual network and attention mechanism identification of rice leaf diseases. Comput Electron Agricult. 2023;204:107543. doi: 10.1016/j.compag.2022.107543

[33] Yang H, Liu Z. Image recognition technology of crop diseases based on neural network model fusion. J Electron Imaging. 2023;32(1):11202. doi: 10.1117/1.JEI.32.1.011202

[34] Yu M, Ma X, Guan H. Recognition method of soybean leaf diseases using residual neural network based on transfer learning. Ecol Inform. 2023;76:102096. doi: 10.1016/j.ecoinf.2023.102096

[35] Wu Q, Ji M, Deng Z. Automatic detection and severity assessment of pepper bacterial spot disease via multimodels based on convolutional neural networks. Int J Agricult Environ Inf Syst. 2020;11(2):29–43. doi: 10.4018/ijaeis.2020040103

[36] Haque I, Islam MdA, Roy K, Rahaman MdM, Shohan AA, Islam MdS. Classifying pepper disease based on transfer learning: a deep learning approach. In: 2022 International Conference on Applied Artificial Intelligence and Computing (ICAAIC). 2022. p. 620–9. 10.1109/icaaic53929.2022.9793178

[37] Mahamud F, Neloy MdAI, Barua P, Das M, Nahar N, Hossain MS, et al. Bell pepper leaf disease classification using convolutional neural network. Lecture Notes in Networks and Systems. Springer; 2022. p. 75–86. 10.1007/978-3-031-19958-5_8

[38] Mathew MP, Elayidom S, Jagathyraj V. Disease classification in bell pepper plants based on deep learning network architecture. In: 2023 2nd International Conference for Innovation in Technology (INOCON). 2023. p. 1–6. 10.1109/inocon57975.2023.10101269

[39] Begum SSA, Syed H. CSIU-Net+: pepper and corn leaves classification and severity identification using hybrid optimization. Environ Res Commun. 2024;6(5):055021. doi: 10.1088/2515-7620/ad4900

[40] Kundu N, Rani G, Dhaka VS, Gupta K, Nayaka SC, Vocaturo E, et al. Disease detection, severity prediction, and crop loss estimation in MaizeCrop using deep learning. Artif Intell Agricult. 2022;6:276–91. doi: 10.1016/j.aiia.2022.11.002

[41] Sibiya M, Sumbwanyambe M. Automatic fuzzy logic-based maize common rust disease severity predictions with thresholding and deep learning. Pathogens. 2021;10(2):131. doi: 10.3390/pathogens10020131 33525312 PMC7912646

[42] Phan H, Ahmad A, Saraswat D. Identification of foliar disease regions on corn leaves using SLIC segmentation and deep learning under uniform background and field conditions. IEEE Access. 2022;10:111985–95. doi: 10.1109/access.2022.3215497

[43] Begum SSA, Syed H. GSAtt-CMNetV3: pepper leaf disease classification using osprey optimization. IEEE Access. 2024;12:32493–506. doi: 10.1109/access.2024.3358833

[44] Divyanth LG, Ahmad A, Saraswat D. A two-stage deep-learning based segmentation model for crop disease quantification based on corn field imagery. Smart Agricul Technol. 2023;3:100108. doi: 10.1016/j.atech.2022.100108

[45] Begum SSA, Syed H. Unsupervised deep learning for plant disease and pest identification: a comprehensive approach. In: 2023 6th International Conference on Recent Trends in Advance Computing (ICRTAC). 2023. 158–66. 10.1109/icrtac59277.2023.10480809

[46] Cui S, Su YL, Duan K, Liu Y. Maize leaf disease classification using CBAM and lightweight Autoencoder network. J Ambient Intell Human Comput. 2022;14(6):7297–307. doi: 10.1007/s12652-022-04438-z

[47] Yu H, Liu J, Chen C, Heidari AA, Zhang Q, Chen H, et al. Corn leaf diseases diagnosis based on K-means clustering and deep learning. IEEE Access. 2021;9:143824–35. doi: 10.1109/access.2021.3120379

[48] Optimizing CNN-YOLOv7 models for pepper leaf disease detection and identification. nano-ntp. 2024;20(A9). doi: 10.62441/nano-ntp.v20is9.94

[49] Akhalifi Y, Subekti A. Bell pepper leaf disease classification using fine-tuned transfer learning. J Elektron dan Telekomun. 2023;23(1):55. doi: 10.55981/jet.546

[50] Divyanth LG, Ahmad A, Saraswat D. A two-stage deep-learning based segmentation model for crop disease quantification based on corn field imagery. Smart Agricult Technol. 2023;3:100108. doi: 10.1016/j.atech.2022.100108

[51] Gole P, Bedi P, Marwaha S, Haque MA, Deb CK. TrIncNet: a lightweight vision transformer network for identification of plant diseases. Front Plant Sci. 2023;14:1221557. doi: 10.3389/fpls.2023.1221557 37575937 PMC10414585

[52] Begum SSA, Syed H. CSIU-Net: pepper and corn leaves classification and severity identification using hybrid optimization. Environ Res Commun. 2024;6(5):055021.

[53] Li G, Wang Y, Zhao Q, Yuan P, Chang B. PMVT: a lightweight vision transformer for plant disease identification on mobile devices. Front Plant Sci. 2023;14:1256773. doi: 10.3389/fpls.2023.1256773 37822342 PMC10562605

[54] Li G, Wang Y, Zhao Q, Yuan P, Chang B. PMVT: a lightweight vision transformer for plant disease identification on mobile devices. Front Plant Sci. 2023;14:1256773. Chen Y, Wang A, Liu Z, Yue J, Zhang E, Li F, et. al. MoSViT: a lightweight vision transformer framework for efficient disease detection via precision attention mechanism. Front Artif Intell. 2025 ;8:1498025.37822342

[55] Li G, Wang Y, Zhao Q, Yuan P, Chang B. PMVT: a lightweight vision transformer for plant disease identification on mobile devices. Front Plant Sci. 2023;14:1256773. doi: 10.3389/fpls.2023.1256773 37822342 PMC10562605

[56] Zhang M, Lin Z, Tang S, Lin C, Zhang L, Dong W, et al. Dual-attention-enhanced MobileViT network: a lightweight model for rice disease identification in field-captured images. Agriculture. 2025;15(6):571.

[57] Mehdipour S, Mirroshandel SA, Tabatabaei SA. Vision transformers in precision agriculture: a comprehensive survey. arXiv preprint 2025. https://arxiv.org/abs/2504.21706

[58] Quan S, Wang J, Jia Z, Xu Q, Yang M. Real-time field disease identification based on a lightweight model. Comput Electron Agricult. 2024;226:109467.

[59] Duhan S, Gulia P, Gill NS, Shukla PK, Khan SB, Almusharraf A, et al. Investigating attention mechanisms for plant disease identification in challenging environments. Heliyon. 2024;10(9):e29802. doi: 10.1016/j.heliyon.2024.e29802 38707335 PMC11066637

[60] Begum AS, Syed H. IDRCNN and BDC-LSTM: an efficient novel ensemble deep learning-based approach for accurate plant disease categorization. Eng Appl Sci Res. 2025;52(1):27–41.

[61] Liu M, Liang H, Hou M. Research on cassava disease classification using the multi-scale fusion model based on EfficientNet and attention mechanism. Front Plant Sci. 2022;13:1088531. doi: 10.3389/fpls.2022.1088531 36618625 PMC9815107

[62] Nigam S, Jain R, Singh VK, Marwaha S, Arora A, Jain S. EfficientNet architecture and attention mechanism-based wheat disease identification model. Procedia Comput Sci. 2024;235:383–93.

[63] Hanh BT, Van Manh H, Nguyen NV. Enhancing the performance of transferred efficientnet models in leaf image-based plant disease classification. J Plant Diseases Protect. 2022;129(3):623–34.

[64] Srivathsan MS, Jenish SA, Arvindhan K, Karthik R. An explainable hybrid feature aggregation network with residual inception positional encoding attention and EfficientNet for cassava leaf disease classification. Sci Rep. 2025;15(1):11750. doi: 10.1038/s41598-025-95985-w 40189680 PMC11973141

[65] Jia L, Wang T, Chen Y, Zang Y, Li X, Shi H, et al. MobileNet-CA-YOLO: an improved YOLOv7 based on the MobileNetV3 and attention mechanism for rice pests and diseases detection. Agriculture. 2023;13(7):1285.

[66] Bi C, Xu S, Hu N, Zhang S, Zhu Z, Yu H. Identification method of corn leaf disease based on improved Mobilenetv3 model. Agronomy. 2023;13(2):300.

[67] Begum SSA, Syed H. GSAtt-CMNetV3: pepper leaf disease classification using osprey optimization. IEEE Access. 2024;12:32493–506. doi: 10.1109/access.2024.3358833

