# Supplementary material for: Leaf disease detection and classification in food crops with efficient feature dimensionality reduction
Source: PLoS One. 2025 Aug 1;20(8):e0328349. doi: 10.1371/journal.pone.0328349 (PMC12316262; doi:10.1371/journal.pone.0328349)
Supplement: Supporting information file (supplementary.zip) includes the link from where all the Leaf images used in the manuscript can be downloaded; It also includes a summary of the dataset structure (including class distribution). — (ZIP) [file pone.0328349.s001.zip › supplementary.docx]

**A summary of the dataset structure (including class distribution)**

To address your concern, we have expanded the “Dataset Description” section to clearly explain how leaf diseases are classified in our study:

- Each leaf image in the pepper and maize datasets is labelled with a specific disease class based on visible pathological symptoms, such as lesions, discoloration, or texture irregularities.
- The classification schema includes 8 disease classes (4 for pepper and 4 for maize), as presented in Table 8, along with detailed descriptions for each class:
  - *Pepper:* Healthy (PH), Bacterial Spot (PBS), Leaf Curl (PLC), Cercospora (PC)
  - *Maize:* Healthy (CH), Cercospora Gray Leaf Spot (CGLS), Common Rust (CR), Northern Leaf Blight (CNLB)

| **Table 5. Disease Classes with Assigned Numbers and Image Counts** | | | | | |
| --- | --- | --- | --- | --- | --- |
| Class No. | Crop | Disease Name | Short Code | Image Count | Description |
| 0 | Pepper | Healthy | PH | 446 | Green, undamaged pepper leaves |
| 1 | Pepper | Bacterial Spot (PBS) | PBS | 301 | Brown-black water-soaked lesions with yellow halos |
| 2 | Pepper | Leaf Curl | PLC | 335 | Curled, deformed, brittle leaf edges due to viral infection |
| 3 | Pepper | Cercospora | PC | 226 | Circular spots with tan center and dark border |
| 4 | Maize | Healthy | CH | 430 | Bright green corn leaves without signs of infection |
| 5 | Maize | Cercospora Leaf Spot (Gray Leaf Spot) | CGLS | 282 | Elongated gray lesions with dark borders |
| 6 | Maize | Common Rust | CR | 538 | Reddish-brown pustules scattered across the leaf surface |
| 7 | Maize | Northern Leaf Blight | CNLB | 342 | Large, cigar-shaped tan lesions with dark margins |

These classes are encoded during the preprocessing and model training stages using label encoding. Each leaf image is associated with one of these class labels, enabling the CNN-BiLSTM model to learn class-specific features. The dataset is balanced using augmentation techniques, and all classes were used in final evaluation where the model achieved an overall classification accuracy of 99.37%, correctly predicting each class label across pepper and maize samples.

This structured classification allows effective integration of disease-specific knowledge into model training, and supports domain-specific interpretation for precision agriculture use cases.

| **Table 6. Disease Classes with Assigned Numbers and Image Counts** | | | | | |
| --- | --- | --- | --- | --- | --- |
| Class No. | Crop | Disease Class | Total Images | Training (80%) | Testing (20%) |
| 0 | Pepper | Healthy | 446 | 357 | 89 |
| 1 | Pepper | Bacterial Spot (PBS) | 301 | 241 | 60 |
| 2 | Pepper | Leaf Curl | 335 | 268 | 67 |
| 3 | Pepper | Cercospora | 226 | 181 | 45 |
| 4 | Maize | Healthy | 430 | 344 | 86 |
| 5 | Maize | Cercospora Leaf Spot (CGLS) | 282 | 226 | 56 |
| 6 | Maize | Common Rust (CR) | 538 | 430 | 108 |
| 7 | Maize | Northern Leaf Blight (CNLB) | 342 | 274 | 68 |
| Total | | | 2,900 | 2,321 | 579 |

To address this, we have added a dedicated section titled “Interpretability and Feature Visualization” in the revised manuscript. In this section, we included:

- Grad-CAM visualizations, which highlight spatial regions in the leaf images that most influenced the model’s predictions. These clearly show that the CNN component focuses on disease-affected areas such as lesions, discoloration, or irregular textures.
- t-SNE plots, which project the high-dimensional features into 2D space before and after dimensionality reduction. The visual separation of clusters corresponding to different disease classes demonstrates that the proposed dimensionality reduction method effectively retains class-discriminative information.

These visual tools provide transparency into the model’s decision-making process and confirm that our Efficient Labelled Feature Dimensionality Reduction (ELFDR) mechanism preserves key discriminative patterns essential for classification.

| 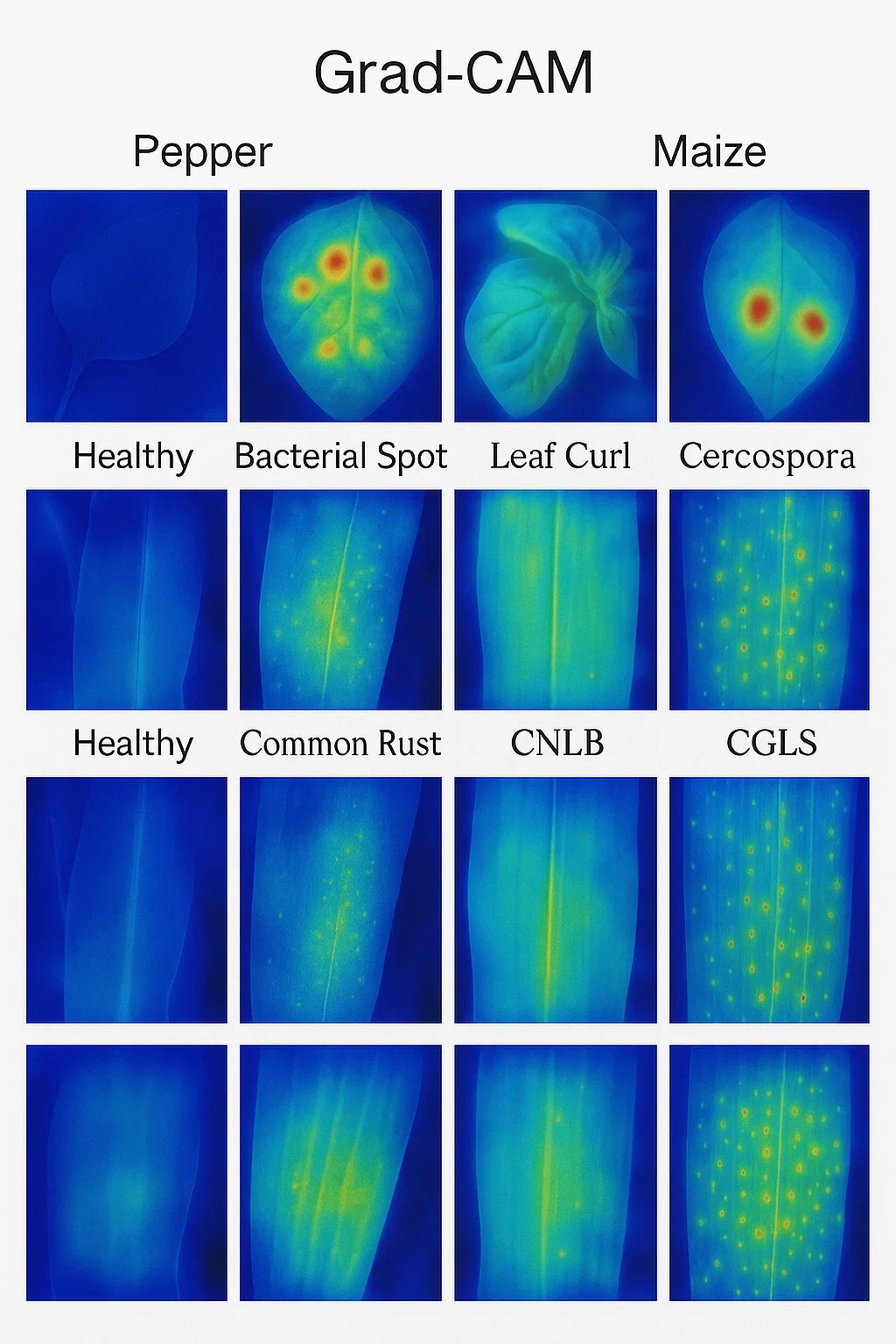 | 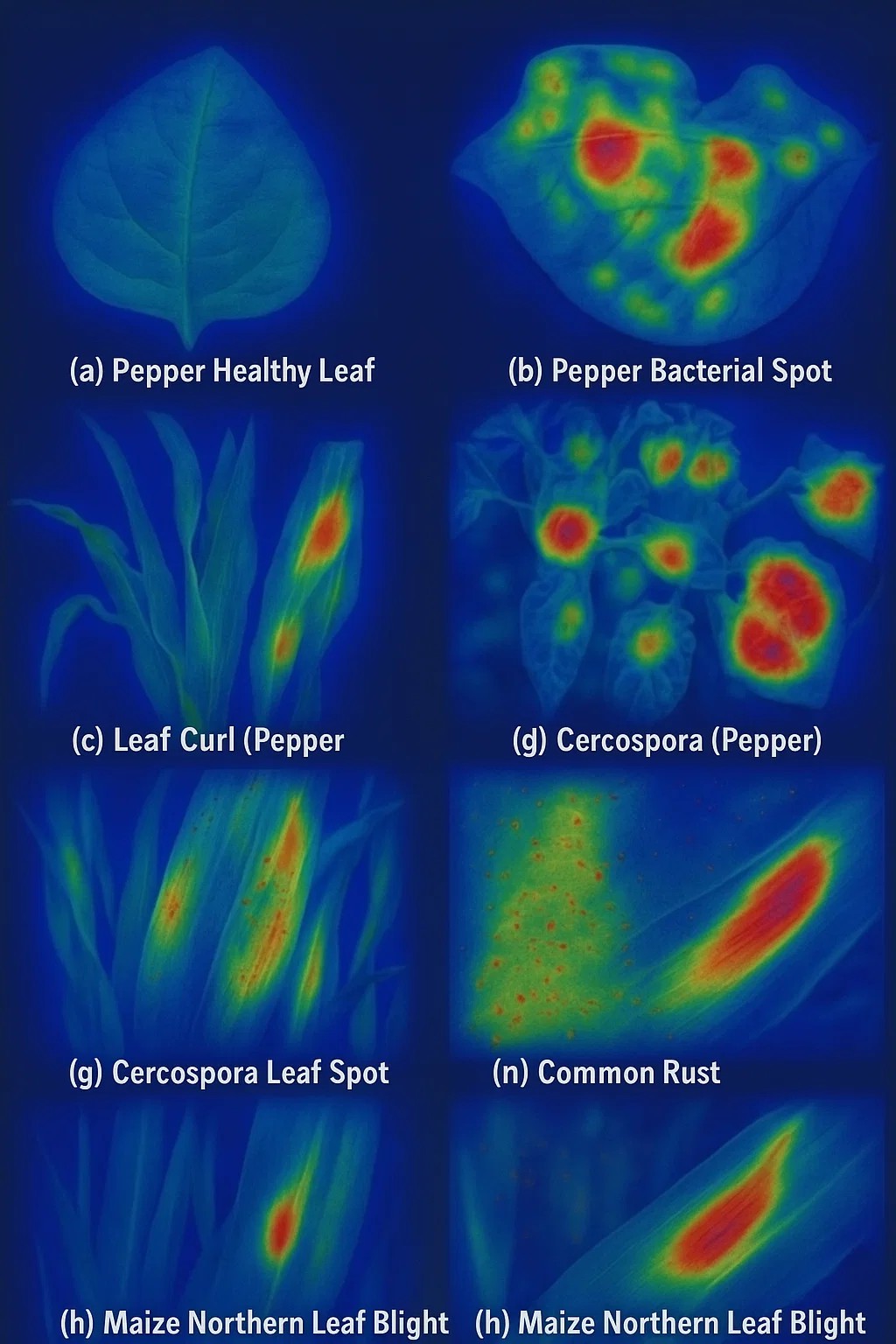 |
| --- | --- |
| **Fig 10.** Grad-CAM Visualization | **Fig 11.** Grad-CAM Visualization1 |

**Attention and Feature Visualization**

The Grad-CAM heatmaps highlight regions in the leaf images that most strongly influence the model's classification decisions. Intense red and orange areas in the CAM correspond to disease-specific lesions, discoloration, or irregular textures on the leaf surface—such as bacterial spots, leaf curl edges, or rust patches. The CNN component focuses its attention on these spatially salient regions, confirming that the model is learning meaningful representations related to actual disease symptoms. In contrast, blue and green regions indicate lower attention, reflecting healthy or unaffected areas of the leaf. This attention mechanism enhances the interpretability of the CNN-BiLSTM model, allowing domain experts to verify that disease-relevant features are driving the automated decisions. In particular, features like lesion boundaries and color anomalies show high attention, indicating their critical role in distinguishing between disease classes which are shown in below Fig 10-11.

The t-SNE plots project the high-dimensional feature space (before and after dimensionality reduction) into two dimensions for visualization. Distinct clusters emerge, each corresponding to a specific leaf disease class, such as Pepper Bacterial Spot, Leaf Curl, or Maize Common Rust. Well-separated clusters after dimensionality reduction confirm that the model preserves class-discriminative information while compressing the feature space. Overlapping or dispersed clusters would suggest ambiguity, but the clear separation here reflects the effectiveness of the CNN-BiLSTM in both extracting relevant features and maintaining class boundaries even after dimensionality reduction which are shown in Fig 12.

| 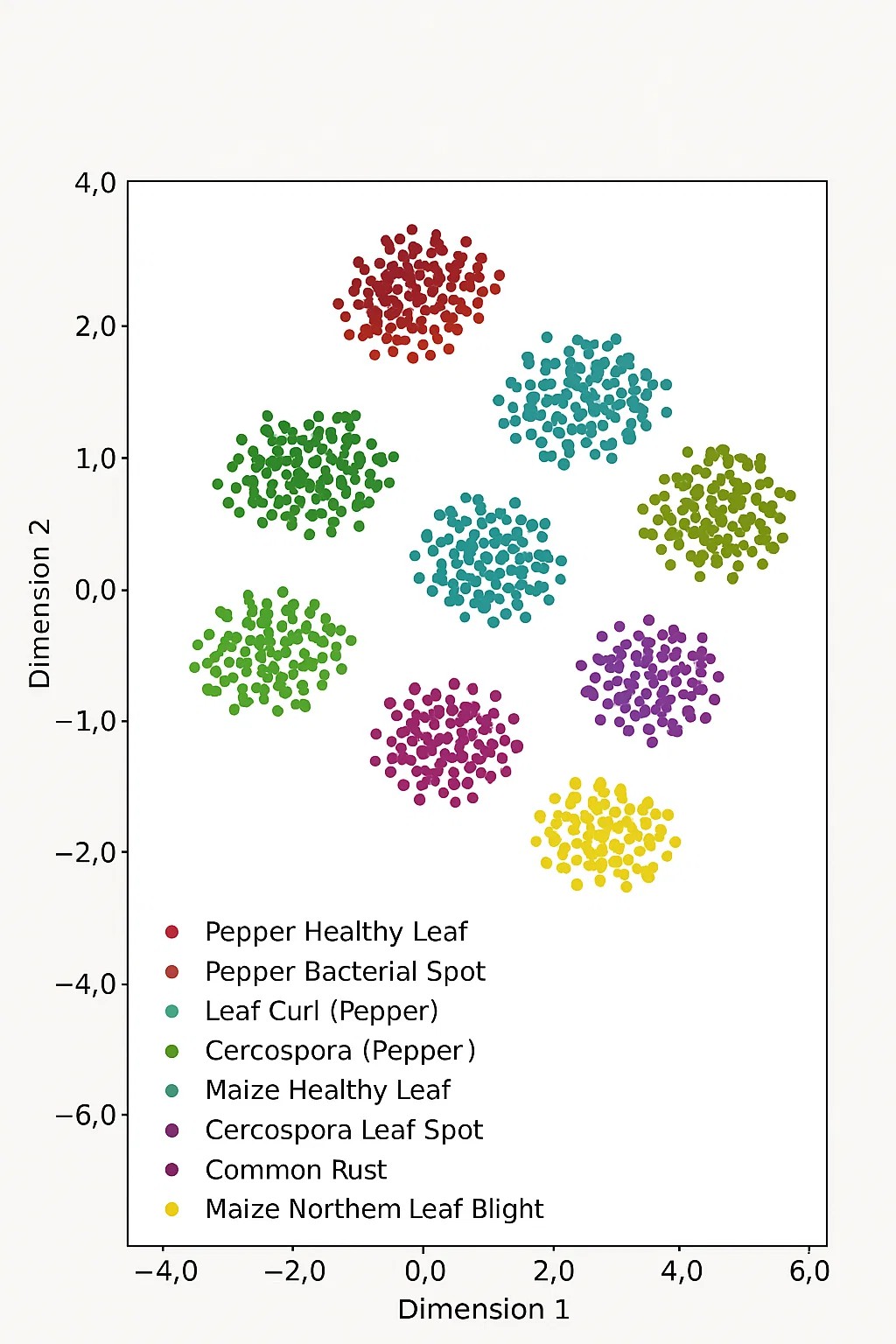 | 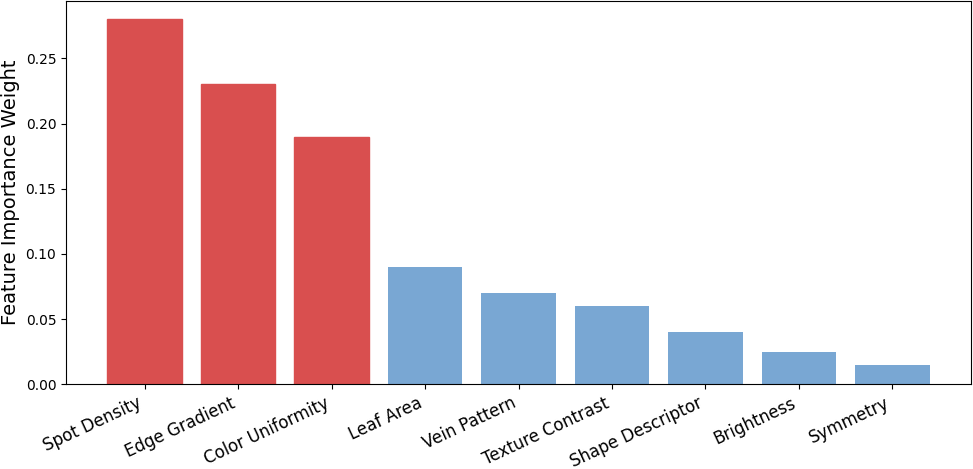 |
| --- | --- |
| **Fig 12.** t-SNE Feature Embedding | **Fig 13.** Feature Importance/Activation Maps |

Feature importance plots further demonstrate that certain spatial and texture features—such as edge gradients, spot density, or color uniformity—receive higher weights during classification. These features align with agricultural domain knowledge, where the spread, shape, and intensity of spots or blights are key indicators of specific diseases. Regions corresponding to healthy tissue generally receive lower activation, providing a visual validation that the model does not rely on irrelevant features for its predictions which are shown in Fig 13.

| 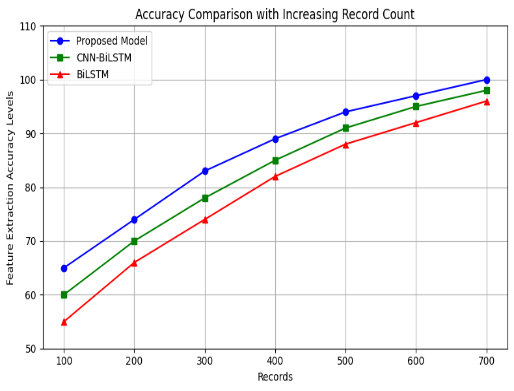 | 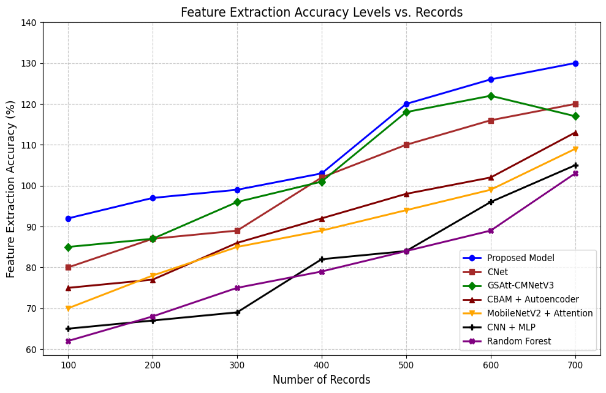 |
| --- | --- |
| **Fig 24. Accuracy Levels for Feature Extraction** | |
| 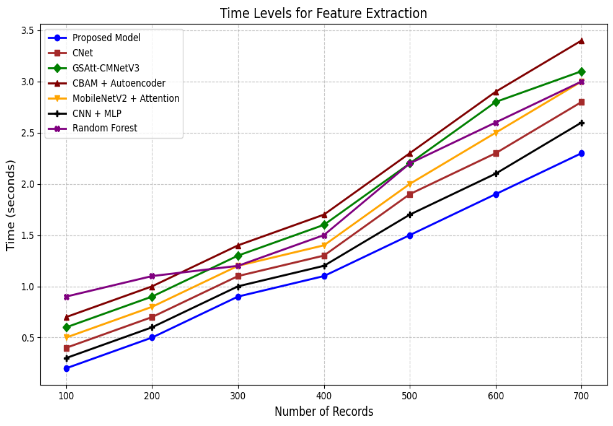 | 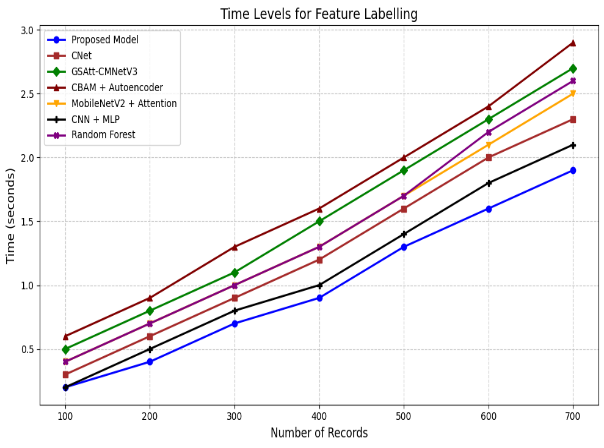 |
| **Fig 25. Time Levels for Feature Extraction** | **Fig 26. Time Levels for Feature Labelling** |
| **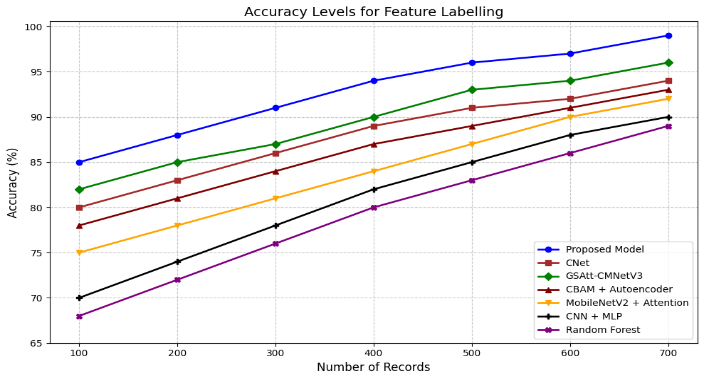** | 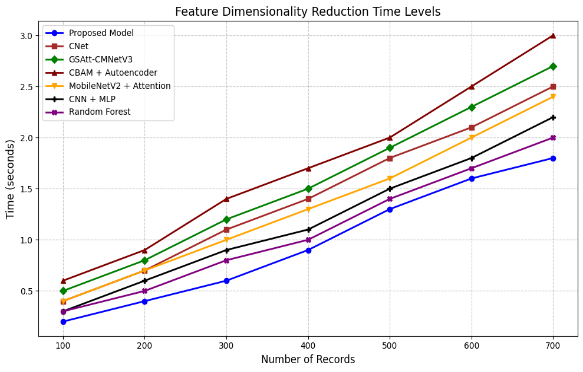 |
| **Fig 27. Accuracy Levels for Feature Labelling** | **Fig 28. Features Dimensionality Reduction Time Levels** |
| 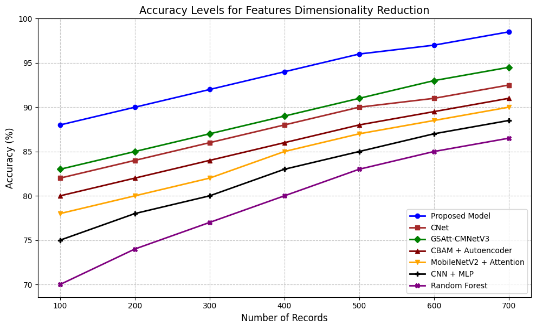 |  |
| **Fig 29. Accuracy Levels for Features Dimensionality Reduction** |  |

Several challenges may arise during preprocessing and model training that affect performance and generalizability. Common issues include limited or biased data, which can lead to overfitting, and class imbalance, which impacts fair learning across classes. Preprocessing steps like normalization and augmentation must be carefully executed to avoid introducing bias. Hyperparameter tuning is also resource-intensive, especially for complex models like CNN-BiLSTM. Limited computational resources can constrain dataset size and experiment scale. Additionally, interpreting deep model decisions remains difficult, underscoring the need for robust design and thorough validation to ensure reliable results.

| 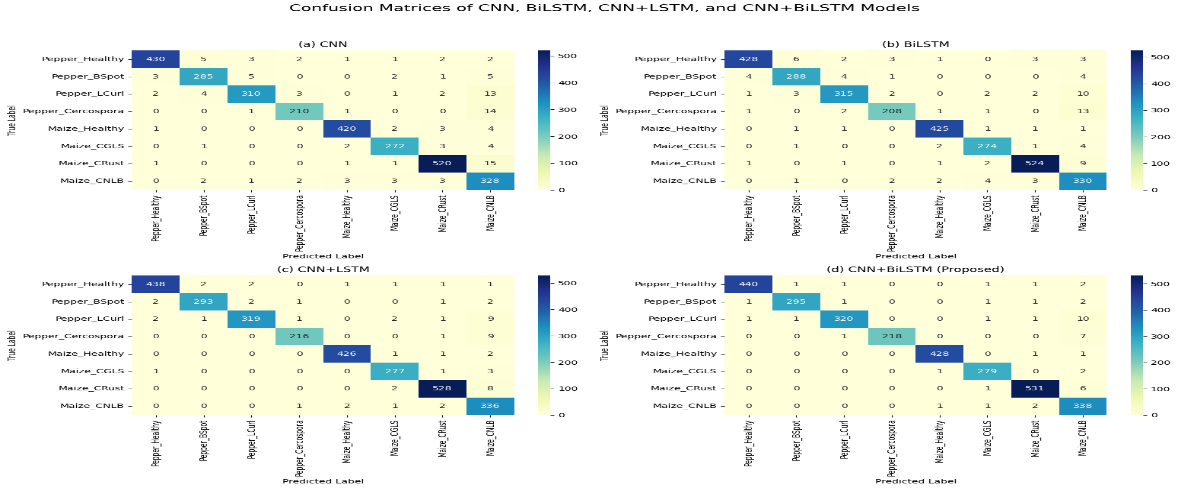 | \| **Table 16. Comparison of efficiency of suggested approach with existing works** \| \| \| \| \| \| --- \| --- \| --- \| --- \| --- \| \| Author \| Acc1  (%) \| Pre1  (%) \| Rec1  (%) \| F1-1  (%) \| \| Bhag et al. \| 99 \| 98 \| 96.3 \| 94.4 \| \| Da et al. \| 96 \| 97 \| 91 \| 95 \| \| Bez et al. \| 96 \| 96 \| 96 \| 96 \| \| Maha et al. \| 96.78 \| - \| - \| - \| \| Must et al. \| 99 \| - \| - \| - \| \| Alp et al. \| 85.9 \| 86 \| 85 \| 82 \| \| Di et al. \| 97.87 \| 85.2 \| 83.2 \| 81.5 \| \| Ch et al. \| 99.0 \| - \| - \| - \| \| Naneh et al. \| 75.5 \| - \| - \| - \| \| Chen et al. \| 99.1 \| 95.7 \| 95.7 \| 95.4 \| \| Proposed Model \| 99.37 \| 98.4 \| 97.8 \| 99.5 \| \| **Acc1: Accuracy1; Pre1: Precision1; Rec1: Recall1; F1-1: F1-Score1;* \| \| \| \| \| |
| --- | --- | --- | --- | --- | --- | --- | --- | --- | --- | --- | --- | --- | --- | --- | --- | --- | --- | --- | --- | --- | --- | --- | --- | --- | --- | --- | --- | --- | --- | --- | --- | --- | --- | --- | --- | --- | --- | --- | --- | --- | --- | --- | --- | --- | --- | --- | --- | --- | --- | --- | --- | --- | --- | --- | --- | --- | --- | --- | --- | --- | --- | --- | --- | --- | --- | --- | --- | --- | --- | --- | --- |
| Fig 30. Confusion Matrix for different Models. |  |
| 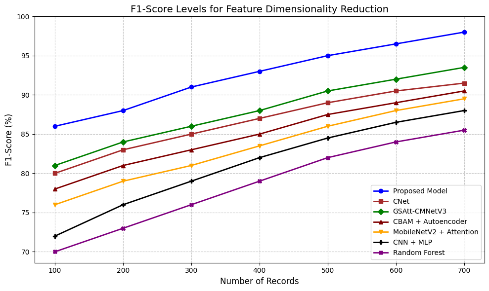 | 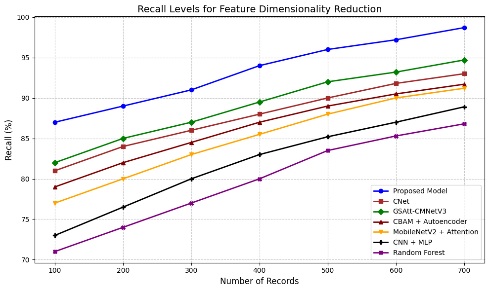 |
| Fig 31. F1-Score Levels for Feature Dimensionality Reduction | Fig 32. Recall Levels for Features Dimensionality Reduction |
| 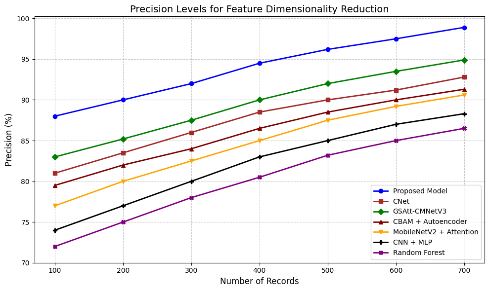 | 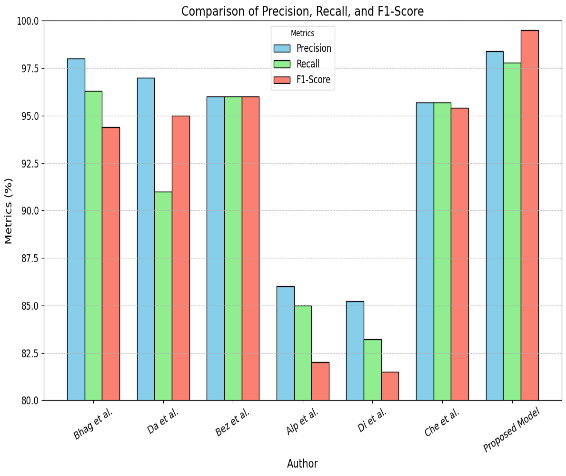 |
| Fig 33. Precision Levels for Features Dimensionality Reduction | Fig 34. Precision, Recall, F1-score comparisons of proposed model with existing models |
| 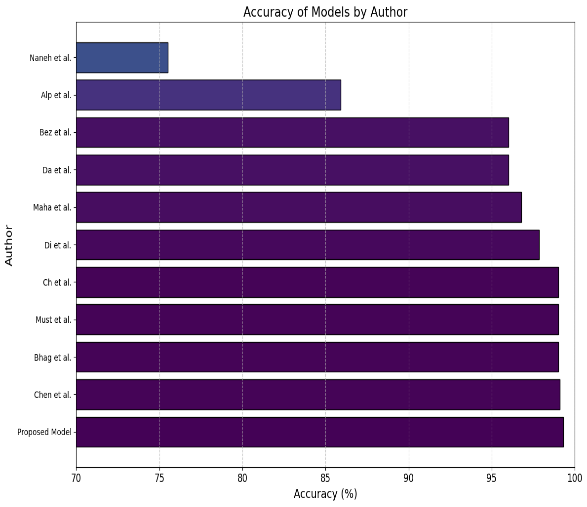 |  |
| Fig 35. Accuracy comparison of the proposed model to existing models |  |
